# An unbiased, sustainable, evidence-informed Universal Food Guide: a timely template for national food guides

**DOI:** 10.1186/s12937-024-01018-z

**Published:** 2024-10-18

**Authors:** Elizabeth Dean, Jia Xu, Alice Yee-Men Jones, Mantana Vongsirinavarat, Constantina Lomi, Pintu Kumar, Etienne Ngeh, Maximilian A. Storz

**Affiliations:** 1 Faculty of Medicine, Department of Physical Therapy, University of British Columbia, Vancouver, BC Canada; 2 Healing Without Medicine, Shenzhen, China; 3 Physicians Committee for Responsible Medicine, Washington, USA; 4 School of Health & Rehabilitation Sciences, The University of Queensland, Brisbane, Australia; 5 Mahidol University, Bangkok, Thailand; 6 Karolinska University Hospital, Stockholm, Sweden; 7 All India Institute of Medical Sciences, New Delhi, India; 8 Louis University Institute, Douala, Cameroon; 9 Research Organisation for Health Education and Rehabilitation, and Guideline International Network African Regional Community, Yaoundé, Cameroon; 10 Department of Internal Medicine II, Centre for Complementary Medicine, Medical Center, and Faculty of Medicine, University of Freiburg, Freiburg, Germany

**Keywords:** Chronic low-grade systemic inflammation, Disease prevention, Health, Non-communicable diseases, Evidence-informed nutrition, Food based dietary guidelines, Population health, Ethics

## Abstract

**Background:**

Although national food guides are designed, ostensibly, to translate scientific evidence with respect to food, dietary patterns, and health, their development has increasingly become a corporate/political process as well as scientific one; often with corporate/political influences overriding science. Our aim was to construct an unbiased, sustainable, evidence-informed Universal Food Guide to serve as a template for countries to develop their unique guides, thereby, provide a valid resource for health professionals, health authorities, and the public.

**Methods:**

To address our aim, we conducted an integrative review of multiple evidence-informed sources (e.g., established databases, evidence syntheses, scholarly treatises, and policy documents) related to four areas: 1. Food guides’ utility and conflicts of interest; 2. The evidence-based healthiest diet; 3. Constituents of the Universal Food Guide template; and 4. Implications for population health; regulation/governance; environment/climate/planetary health; and ethics.

**Results:**

The eating pattern that is healthiest for humans (i.e., most natural, and associated with maximal health across the life cycle; reduced non-communicable disease (NCD) risk; and minimal end-of-life illness) is whole food, low fat, plant-based, especially vegan, with the absence of ultra-processed food. Disparities in national food guide recommendations can be explained by factors other than science, specifically, corporate/political interests reflected in heavily government-subsidized, animal-sourced products; and trends toward dominance of daily consumption of processed/ultra-processed foods. Both trends have well-documented adverse consequences, i.e., NCDs and endangered environmental/planetary health. Commitment to an evidence-informed plant-based eating pattern, particularly vegan, will reduce risks/manifestations of NCDs; inform healthy food and nutrition policy regulation/governance; support sustainable environment/climate and planetary health; and is ethical with respect to ‘best’ evidence-based practice, and human and animal welfare.

**Conclusion:**

The Universal Food Guide that serves as a template for national food guides is both urgent and timely given the well-documented health-harming influences that corporate stakeholders/politicians and advisory committees with conflicts of interest, exert on national food guides. Such influence contributes to the largely-preventable NCDs and environmental issues. Policy makers, health professionals, and the public need unbiased, scientific evidence as informed by the Universal Food Guide, to inform their recommendations and choices.

**Supplementary Information:**

The online version contains supplementary material available at 10.1186/s12937-024-01018-z.

## Introduction

In 2019, the EAT Lancet Commission reported on the timely adoption of healthy diets sourced from sustainable food systems [1, 2]. A disparity exists between the scientific evidence reflected in the Report’s recommendations and dietary trends worldwide, most notably the ubiquitous consumption of the Standard Western or American Diet (SWAD) [3]. This disparity and recent arguments not supporting the recommendations of the Report warrant reconciling, with a view to align national food guidelines with the science. Factors contributing to this disparity include lack of awareness of the healthfulness of legumes by the public [4]; micronutrient deficiencies [5]; and, contrary to the evidence, lack of acknowledgement by the World Health Organization that animal-sourced foods may adversely affect health [6].

Of the six pillars of lifestyle medicine, nutrition is inarguably the foundation of health [7–20]. Diet-related non-communicable diseases (NCDs) have been well documented and include cardiovascular disease, several cancers, hypertension, stroke, obesity, type 2 diabetes mellitus, metabolic syndrome, non-alcoholic fatty liver disease, renal disease, dementia, and Alzheimer’s disease [21–47]. In the United States, low quality, obesogenic diets now surpass smoking as the leading cause of premature death [48]. As a priority, population-level as well as individual-level interventions have been called for, to improve population health and reduce NCDs [21, 36, 49]. In turn, such interventions, if evidence-based, will reduce chronic disease health care costs that have become unsustainable [50].

Supported by other sources, the Diet Collaborators responsible for the Global Burden of Disease position statement, reported that 11 million deaths globally and 255 million disability-adjusted life-years were attributable to dietary risk factors such as high intake of sodium, low intake of whole grains, and low intake of fruits [42, 51, 52]. Increasingly, more people are now dying prematurely of heart disease, for example, in low- and middle-income countries and the problem has become particularly serious among the poor [53].

Since the end of World War 2 in 1945, the global economy has grown exponentially. In parallel, two nutrition-related trends have emerged [54]. These trends include: 1. the escalated consumption of animal-sourced products and corresponding increase in industrialized factory farming [55]; and, 2. the consumption of processed and ultra-processed food (UPF) that has evolved with sophisticated engineering, production, and marketing strategies [56–58].

These two trends constitute the ‘commercial determinants of health’ [59] and characterize the unhealthy, calorie-dense, nutrient-poor SWAD [33, 60–62]. Although the prevalent omnivorous SWAD has become viewed as normative, i.e., ‘natural, normal, and necessary’ [63–65], this is not supported by the evidence (Table 1, Supplemental Materials, Animal-sourced foods and evidence supporting associated health risks).

Food processing has had a role in improving food safety and distribution, however, much UPF has support only for its being harmful to health, with no support for its being healthful [66]. Further, qualifiers such as ‘limit’, ‘reduce’ or ‘eat in moderation’ are typically attached to unhealthy foods, not only in the category of discretionary calories, e.g., those high in saturated fat, sugar, and salt; but also UPF. Such terms selectively used in reference to unhealthy foods in food guides, normalize unhealthy choices [67].

The impact of these escalating trends on human health, specifically the nutrition-related NCDs, is now well documented and constitutes a global crisis [68–70]. Only in more recent decades has their impact on global environmental destruction and the climate crisis been documented [71–74].

Commensurate with these global trends over the past 40 to 60 years, governments began recognizing the unequivocal link between nutrition and health, and national food guides began to emerge. These guides were designed to serve as tools to inform the general public, health professionals, and health policy makers, about healthy eating patterns based on the scientific evidence and expert consensus [75]. Periodically, these guides are updated, ostensibly to reflect evolving nutrition science.

The need for strictly evidence-based food guides is further reflected by the health care economic burden of nutrition-related NCDs. This burden is related to the adverse dietary trends of consumption of animal-sourced foods and UPF as described; both of which have reached unsustainable proportions [76, 77]. Based on one sophisticated econometric analysis, Scarborough and colleagues reported that, of the various lifestyle factors, low quality nutrition was the leading contributor to health care costs in the United Kingdom [78]. This finding is not dissimilar to trends in other western countries, and is increasingly so in low- and middle-income countries [79, 80]. The economic burden of inadequate consumption of vegetables and fruit has been documented in Canada [81] and the United States [48, 62, 82], as well as in Sweden [83], to name a few countries. Based on a sophisticated modelling study in Sweden, Saha and colleagues [83] reported that if Swedes adhered to evidence-based guidelines regarding the intake of vegetables and fruit and of fiber, 47% of cardiovascular and cancer deaths could be prevented. Further, based on data from the 2019 Global Burden of Disease study involving 204 countries and territories, Chong and colleagues [84] compared trends in disability-adjusted life-years and deaths, and obesity and malnutrition over 20 years (2000 to 2019). They concluded that the progressively increasing obesity burden would be accentuated further in the decades ahead, comparable to the malnutrition burden in many countries.

Our overarching aim was to construct an unbiased, sustainable, evidence-informed Universal Food Guide to serve as a template for countries to develop their unique guides. Such a guide would provide a valid resource for the public health authorities, health professionals, and the public in order to make informed decisions with respect to their health and that of their families. To address this aim, we conducted an integrative review of multiple evidence-informed sources with key words related to four areas: 1. Food guides’ utility and conflicts of interest; 2. The evidence-based healthiest diet; 3. Constituents of the Universal Food Guide template; and 4. Implications for population health; regulation and governance; environment/climate and planetary health; and ethics. This comprehensive integrative review consolidates the breadth of the evidence to date related to the health benefits and reduced harms of plant-based nutrition, particularly vegan nutrition, in the interest of advancing global health through nutritional science.

## Methods

Our aim met the criteria for conducting an integrative review, e.g., provide a comprehensive overview of a wide range of sources and literature on a particular subject; integrate findings from diverse methodologies; synthesize existing research on a topic to generate new insights and provide a comprehensive understanding; synthesize evidence to inform policy, clinical guidelines, and best practices by bringing together findings from diverse sources to provide clear, evidence-based recommendations; and to track the development of a topic over time, showing how understanding or approaches have evolved; and to help identify and explain disparities [85, 86].

We used established methods [85, 86] to capture a diverse range of resources including empirical studies, evidence syntheses, and reviews across electronic databases, an integrative review was conducted based on key words related to the four areas of interest above, database searches of PubMed, EMBASE, CINAHL, Web of Science, Google Scholar, the Cochrane Database of Systematic Reviews, and institutional repositories*, *in addition to published monographs of evidence syntheses based on peer-reviewed sources, government publications, policy documents, scholarly monographs and treatises, documentaries based on scientific evidence, and credible journalistic sources including historic film footage. Iteratively, citations from retrieved articles were examined to extend the breadth of the review and for cross-referencing.

## Results

### Food guides’ utility and conflicts of interest

#### Emergence of the industrialized western diet and food guides

Over millennia, as humans migrated, they needed to be resourceful for survival. They adapted their diets to their environments and became opportunistic omnivores. Such adaptations were out of necessity for survival, rather than being ideal nutritionally [87, 88].

The year 1945 was a turning point in the emergence of the industrialized western diet. Up until the end of World War 2 in 1945, dietary patterns around the world largely reflected the consumption of local foods that were grown close to home, thus readily available [87]. After World War 2, the economic and technologic dominance of the west, particularly the United States, dramatically influenced cultures worldwide including their food systems [86–89]. Since then, lifestyle choices including available dietary options and choices, have been increasingly influenced by sociocultural conditioning and sophisticated corporate food promotion and advertising, rather than by millions of years of evolutionary ancestry and emerging science [87, 88].

The SWAD has been reported to be pro-oxidative and pro-inflammatory, the consequence of several characteristics that are highly deleterious to human health, e.g., high glycemic load; high fatty acid composition; poor macronutrient composition and quality; low micronutrient density; increased renal acid load and altered acid–base balance; reversal of the potassium-sodium ratio (to high sodium and low potassium); and pathologically low fiber content (characterized by low consumption of legumes, whole grains, vegetables and fruit) [88–90].

The pathological consequences of the consumption of this pro-inflammatory eating pattern since childhood, are cumulative and manifest in nutrition-related NCDs later in life [1]. Chronic oxidative stress and low-grade systemic inflammation (CLGSI) associated with SWAD is the common pathway for NCDs and their related premature death [91–94]. These NCDs are strongly associated with both the consumption of animal-sourced foods and UPF [3, 95–100]. Overweight and obesity are common correlates with other NCDs and their underlying CLGSI, for which, a healthy diet to restore metabolic health rather than weight loss interventions per se, is effective and more sustainable [101, 102]. Given that NCDs emerge from a common inflammatory pathway explains why they cluster (multi-morbidity) and that the single-disease framework to address them, is no longer tenable [103, 104].

A second turning point with respect to nutrition recommendations had its epicenter in the United States, early in 1977. Divergence of food guides from the science by corporate and political interests, can be traced back to that country and time [105]. That year, Senator George McGovern and his food advisory committee published the first dietary goals in response to the increasing prevalence of chronic disease (heart disease and diabetes) in the United States [106]. Thus, the first edition of the American food goals strongly recommended decreasing the consumption of meat, dairy, and eggs to reduce saturated fat, as well as reducing salt and sugar intake. Lobbyists reacted vehemently and succeeded in having the wording in the report changed to increase the consumption of lean meat, specifically, ‘choose meat, poultry, and fish which will reduce saturated fat intake’ [107, 108]. Thus, 1977 was the year in which protecting public health in the United States became secondary to protecting corporate health and wealth [107–109]. Since then, food guides began to emerge worldwide that reflected the American food guidelines which had yielded to corporate lobbying and pressure [110, 111], rather than to emerging science. The strength of this western influence on diets in others parts of the world became apparent as healthier traditional diets began being replaced with constituents of the SWAD and nutrition-related NCDs increased correspondingly [85, 87, 112, 113].

#### ‘Commercial determinants of health’ and impact on food guides

The narrative underlying the belief that the consumption of animal-sourced foods is necessary for health, has been promoted by governments with politico-industrial ties and ‘big food’ industries, rather than being informed by science [114–118]. There is no evidence to support that consuming animal-based eating pattern is necessary or healthier than consuming a whole-food, low-fat, plant-based eating pattern, in fact, the contrary. Table 1 (Supplemental Materials; Animal-sourced foods and evidence supporting associated health risks) summarizes evidence related to health harms and disease risks associated with consuming animal-sourced foods, e.g., cardiovascular disease, several cancers, stroke, hypertension, type 2 diabetes mellitus, obesity, metabolic syndrome, gastrointestinal diseases, immune dysfunction, dementia, and Alzheimer’s disease; and premature death.

In addition, over the past several decades [60, 119, 120], excessive food processing has been declared a leading public health threat [121]. Fifteen years ago, Monteiro [121] argued that the extent and purpose of food processing has changed globally. Further, they argue that these changes have driven the emergence of a harmful global food system, and the pandemic of nutrition-related NCDs and premature death [57, 68, 119–146]. In response, he and his group developed the NOVA food classification system to categorize food processing [147, 148]. NOVA has been endorsed by the United Nations and the Pan American Health Organization (Table 1). The ‘golden rule’ that emerged from that classification system was always to select natural or minimally processed foods and opt for freshly made dishes and meals, over ultra-processed products [147]. Although Nova Classification 4 UPF is to be avoided, constituents commonly used to process food (NOVA Classification 2) have documented health-harming risks as well, i.e., oils, fats, sodium and salt, and sugar (Table 2) (Supplemental Materials, Constituents (other than synthetic and other additives) commonly found in processed and ultra-processed foods (NOVA Classifications 2 and 3) and documented health risks).

**Table 1:** Descriptions of the NOVA system for classifying processed and ultra-processed food (‘UPF’)^a^

NOVA Group 1	Consists of unprocessed or minimally processed foods as the basis of the diet.
NOVA Group 2	Contains ‘culinary ingredients’, such as salt, oil, sugar, or starch, which are produced from NOVA1 foods.
NOVA Group 3	Contains ‘processed foods’, such as freshly baked breads, canned vegetables, or cured meats, which are obtained by combining NOVA1 and NOVA2 foods.
NOVA Group 4	Contains ‘ultra-processed foods’ namely ready-to-eat industrially formulated products that are ‘made mostly or entirely from substances derived from foods and additives, with little if any intact Group 1 food’; ultra-processed foods including frozen pizza, soda, fast food, sweets, salty snacks, canned soup, and most breakfast cereals should be avoided.

^a^For more detail and specific definitions and examples of processed and ultra-processed foods, see [98, 147, 148, 152]

The principal ingredients of ‘UPF’ are genetically-engineered foods such as maize, soy and sugar beets which also enter the food chain as animal feed. The European Union has stricter regulations about genetically-modified foods than North America, given questions have been raised by the public about their health consequences and ‘the public’s right to know’. See ‘GMO’s. The Amount of Corn and Soy in the EU’. European Parliament available at https://www.europarl.europa.eu/topics/en/article/20151013STO97392/eight-things-you-should-know-about-gmos#:~:text=Are%20GMOs%20allowed%20in%20the,in%20the%20EU%20so%20far; retrieved 6 September 2024. The best way to avoid genetically-engineered foods is to buy !00% certified organic products, whole foods and fresh produce

UPF has been engineered for convenience, low cost, hyper-palatability, crave-ability (addictive qualities) and unnaturally long shelf lives [66]. Their ‘..ultraprocessing makes them highly profitable, intensely appealing and intrinsically unhealthy’ [121]. Industrialized UPF is designed for sustainable profits rather than the sustainable health of populations [66, 149–154]. The SWAD has become increasingly dominated by processed foods, particularly UPF [152]. Across nations, up to 80% of people’s calories are derived from UPF [153, 154].

UPF that is calorie-dense and nutrient-poor has been reported to be formulated with cheap commodity crops and refined bleached deodorized fats/oils, modified starches, and protein isolates [66]. These then are often treated with additives, emulsifiers, stabilizers, preservatives, flavorings, colorants, and taste enhancers [66]. One major public health concern that affects children, is that synthetic compounds in UPF can be endocrine-disruptors adversely affecting normal development [155, 156]. The Center for Science in the Public Interest (CSPI) has long protested the lack of oversight and regulation of ingredients permitted in UPF, under the guise of GRAS (Generally Recognized As Safe). Recently, the CSPI has drawn public attention to the unregulated ingredients simply termed as ‘flavors’ and ‘spices’ which are not specifically identified [157], thus the consumer cannot establish precisely what they are consuming.

Across high- to low-income countries, unidentified or ambiguously labelled ingredients in food products are out of direct control of individuals. Inadequate labelling can obscure the products’ actual ingredients. Excess intake of dietary salt, for example, is one of the leading risks to global health and incidence of diet-related NCDs. People may be less aware of sodium and salt that is added during food processing rather than added at the table [158].

UPF is heavily marketed to vulnerable populations including children and marginalized groups [159–163]. As UPF markets become saturated in the west, these industries are shifting their sights to potentially more profitable markets in low- and middle-income countries such as African countries and India [113]. As people in these countries increasingly shift from their traditional eating patterns [93, 164], these countries are now faced with a double disease burden, i.e., under-nutrition, and over-nutrition that is secondary to SWAD that is prevalent in western countries [165–167].

#### Factors compromising valid knowledge translation into food guides

The purported objective of national food guides is to provide the best evidence-based information about dietary constituents that promote children’s growth and development and enable adults to thrive across the life cycle; are associated reduced nutrition-related NCDs; and promote a long healthy life with little-to-no end-of-life illness [75, 168–170]. Because food guides are often influenced by stakeholders with vested interests, and public health authorities with often undisclosed corporate/political ties, as in the United States [171], public trust is undermined [172].

One national dietary guideline that claims to be more operationally committed to evidence as opposed to corporate interests, is the 2019 Canada Food Guide [173]. In this guide, food groups are illustrated proportionally on the plate across meals, rather than serving sizes. Dai and colleagues analyzed the 2019 version vs. the previous 2007 version, regarding its greater transparency and being more evidence-based. They advocated that a method criteria approach is needed in the next iteration to incorporate priorities such as food sustainability [174].

Several other factors may explain the selective and inadequate translation of nutrition knowledge into national food guides. These include variations in the definitions of plant-based nutrition [175, 176]; nutrition has not been prioritized in the curricula of health professionals’ education [18, 177]; nutrition studies may benefit from using the whole-food, low-fat vegan diet as a reference [101, 178]; and randomized controlled trials are not necessarily optimal research designs to evaluate eating patterns [179–183]. Comparable to investigating the effects of smoking with randomized controlled trials, this design has comparable ethical limitations in evaluating certain other lifestyle practices such as eating patterns.

Commercial interests have perpetuated the myth that whole-food, low-fat vegan nutrition is unbalanced [184–186]. Although the SWAD is an omnivorous diet to which people have become accustomed socially and culturally, and have acquired a taste for, its health outcomes have been reported to be inferior to a whole-food, low-fat vegan eating pattern even when both are designed to have all the essential nutrients [187].

#### National food guides’ objectives and global influence of the American food guidelines

America’s global influence extends to food systems given the growing westernization of food systems, worldwide. Nations’ traditional, often healthier food systems are being eroded, and diet-related NCDs are escalating globally [3]. For this reason, the Dietary Guidelines for Americans (DGA) are of global interest, notwithstanding the fact that the health of Americans, of which diet is a major contributor, rates the lowest across comparable industrialized countries [188].

The 2020-2025 edition of DGA claims to be grounded in robust scientific reviews of key nutrition and health topics, for each life stage [189]. Despite claims of greater transparency and public participation, there are marked discrepancies between several recommendations in the DGA and the evidence (Table 3, Supplemental Materials, Evidence supporting health-harming risks of The Dietary Guidelines for Americans (DGA)). According to the United States Department of Agriculture, Americans derive 32% of their daily calories from animal-sourced foods; 57% from processed foods; and only 11% from grains, beans, fruits, vegetables, and nuts [190]. This pattern is consistent with a pattern of low nutrient density from fresh and unprocessed foods.

Today, contemporary national food guides including the DGA emphasize fruit and vegetables [75] supporting that plant-based nutrition has been acknowledged by health professionals to be ‘safe and effective’ [184, 191, 192]. The differences in national food guides however, appear to be largely related to food sources/products with strong corporate interest and influence. These include milk and dairy products, meat and derivatives, fish, eggs, and oils [75]. Other differences reflect variations in political pressure for sustainable diets as well as consideration of socio-ethnic and cultural differences [75, 169]. Although most national food guides acknowledge they are based on evidence, often there are subjective qualifications, e.g., ‘*what people prefer*’ [189] and ‘*The guidelines were developed with food habits and culinary culture in mind and backed by scientific evidence*’ [193]. D*e facto*, food guides are to serve the public by informing them about healthy nutrition rather than serve as a platform for ‘big food’ corporations, or for advancing the perceptions and preferences of food guide advisory committee members.

The use of ambiguous language in the DGA conveys more legitimacy to its recommendations than is warranted based on the science [189]. For example, they state ‘Limit foods and beverages higher in added sugars, saturated fat, and sodium, and limit alcoholic beverages’. Qualifiers such as ‘Limit’ are ambiguous. The DGA acknowledge that ‘a healthy dietary pattern doesn’t have much room for extra added sugars, saturated fat, or sodium—or for alcoholic beverages’, yet goes on to state that ‘a small amount of added sugars, saturated fat, or sodium can be added to nutrient-dense foods and beverages to help meet food group recommendations, but foods and beverages high in these components should be limited’. These qualifiers are contradictory and open to interpretation.

Wording of food guides needs to be clear to the average person. With respect to the DGA, we propose that most people would not know how to calculate ‘Less than 10% of calories per day’ for sugar consumption; ‘Less than 10% of calories per day for saturated fat’; and ‘Less than 2,300 mg per day for sodium.

If an adult chooses to drink alcohol, the DGA recommendation of ‘limiting intake to 2 drinks or less in a day for men and 1 drink or less in a day for women’ may have no basis. Given that no safe limit for alcohol consumption has been reported, the 2023 Canadian guidelines advise no more than 1–2 standard alcoholic drinks a week to avoid alcohol-related consequences to oneself or others [194].

In relation to the DGA, recommendation to ‘customize and enjoy nutrient dense food and beverage choices to reflect personal preferences, cultural traditions, and budgetary considerations’ is laudable. However, these recommendations need to be consistent with those that support maximal health.

### The healthiest evidence-based diet

Multiple lines of evidence support that not only is a vegetarian eating pattern optimal for humans, but whole-food, low-fat vegan nutrition with the absence of animal-sourced foods and UPF in the diet, is associated with maximal health outcomes for people and for the planet (i.e., the least carbon footprint, air pollution, and soil and water erosion) (Table 4, Supplemental Materials, Evidence supporting the Universal Food Guide consisting of whole-food, low-fat vegan nutrition; such nutrition is rich in fiber, micronutrients, vitamins and minerals, legumes (beans, peas, and lentils that are all rich protein), whole grains, nuts, and vegetables and fruit; and with little to no added sugar, salt and fat, particularly no trans nor saturated fat). To date, no other eating pattern has been reported that equals or surpasses the health protective effects of such nutrition, in terms of preventing, managing, and often reversing nutrition-related NCDs based on objective outcomes [195–204]; and protecting the health of the planet (See Discussion, section Environment, Climate and Planetary Health).

Table 4 (Supplemental Materials, Evidence supporting the Universal Food Guide consisting of whole-food, low-fat vegan nutrition; such nutrition is rich in fiber, micronutrients, vitamins and minerals, legumes (beans, peas, and lentils that are all rich protein), whole grains, nuts, and vegetables and fruit; and with little to no added sugar, salt and fat, particularly no trans nor saturated fat), lists key lines of evidence supporting that humans are vegan-by-design [205]. Comparative analyses of human anatomy, physiology and metabolism, with carnivorous and particularly omnivorous species, show that humans are dissimilar to natural omnivores on every dimension (Table 4, Supplemental Materials, Evidence supporting the Universal Food Guide consisting of whole-food, low-fat vegan nutrition; such nutrition is rich in fiber, micronutrients, vitamins and minerals, legumes (beans, peas, and lentils that are all rich protein), whole grains, nuts, and vegetables and fruit; and with little to no added sugar, salt and fat, particularly no trans nor saturated fat). Further, evidence from the most comprehensive nutrition study in the world, The China Study [206, 207], supports the health benefits of plant-based nutrition, as does evidence from the long-living residents in the Blue Zones of the world [208, 209]. In addition to the multi-system benefits of plant-based nutrition that have been well established [210, 211], the greater adherence to well-balanced plant-based nutrition, the greater health benefit without any documented harm (Table 4, Supplemental Materials, Evidence supporting the Universal Food Guide consisting of whole-food, low-fat vegan nutrition; such nutrition is rich in fiber, micronutrients, vitamins and minerals, legumes (beans, peas, and lentils that are all rich protein), whole grains, nuts, and vegetables and fruit; and with little to no added sugar, salt and fat, particularly no trans nor saturated fat). Conversely, the less adherence, coupled with the consumption of meat, poultry, dairy, eggs, fish and seafood, the greater potential for their documented health risks and harms (Table 1, Supplemental Materials, Animal-sourced foods and evidence supporting associated health risks).

With respect to diet and NCD reversal, a whole-food, low-fat vegan eating pattern is the only eating pattern that has been reported objectively to reverse coronary atherosclerosis [195, 196]. Over 30 years ago, this was reported in the seminal work of Ornish and colleagues, based on angiographic evidence, in conjunction with exercise [195]. Sub-optimal nutrition associated with the SWAD and its underlying CLGSI has been implicated as a cause or major contributor to multiple chronic NCDs [13, 212–230] as well as heart disease. In addition to being better managed with plant-based nutrition, these diseases can often be reversed with plant-based nutrition, which strongly supports causation [196].

A range of alternative eating patterns, e.g., Atkins, paleo and keto diets, can manipulate human metabolism to produce short-term metabolic and weight loss benefit [231]. These eating patterns have captured the public’s attention, yet are not associated with the benefits of whole-food, low-fat, entirely plant-based nutrition with respect to overall health long-term, and reduced CLGSI, in turn, reduced NCD risk factors or NCD reversal. Adoption of such unnatural diets has been cautioned given their health and disease risks [231]*.*

Based on a sophisticated cross-over research design, the whole-food, low-fat vegan eating pattern was reported to surpass the Mediterranean diet with respect to cardiovascular and metabolic outcomes [232]. Although blood pressure was reduced in both interventions, the Mediterranean diet reduced blood pressure somewhat more. Given the sophistication of this study’s research design, the whole-food, low-fat vegan eating pattern could justifiably serve as a new reference healthy eating pattern, i.e., the ‘gold dietary standard’, against which other dietary patterns could be compared.

#### The Mediterranean diet

Although not a food guide per se, over several decades, the Mediterranean diet has been regarded as the healthiest in the world [233–237]. Thus, its principles and/or constituents have been incorporated into food guides to varying degrees, e.g., abundant vegetables and fruits, legumes, fish, olive oil, and little meat [238, 239]. The basic constituents of the Mediterranean diet are also common to the traditional Chinese diet [206, 207] and to those diets reported in the five Blue Zones of the world [208]. Constituents that are common to the traditional Chinese diet and diets of people in the Blue Zones include a wide variety of vegetables, fruits, whole grains (especially cereal grains, rice and noodles), legumes, nuts, and seeds. Meat and fish are consumed in small quantities compared to Western diets. Both diets include fresh herbs and spices.

The Blue Zones are characterized as having the world’s healthiest and longest living people [208, 240]. Two of the five Blue Zones are in Mediterranean countries, namely, Greece (Ikaria) and Italy (Sardinia). As people in the Blue Zones adopt a more western eating pattern however, their traditional diets have become less protective and NCDs have become more prevalent [241–244]. Similarly, this was reported in two regions in China investigated over 20 years. Over this timeframe the diagnostic features of metabolic syndrome emerged [245, 246].

People consuming plant-based nutrition such as the Mediterranean diet are healthier, and age with less concomitant illness and disability than those consuming the SWAD [247]. Individuals with cancer have been reported to benefit from the Mediterranean diet [248] and to respond more favorably to chemotherapy [249]. This could be explained by the low inflammatory load of the plant-based eating pattern known to reduce oxidative stress and CLGSI associated with NCDs [250–254], and to augment mitochondrial function [255].

Olive oil, a staple of the Mediterranean diet, has been reported to have vascular protective properties [256–259]; an important attribute given endothelial dysfunction is an early feature of atherosclerosis. However, when the effects of olive, soybean and palm oils intake are compared, they exhibited comparable acute detrimental effect over the endothelial function [260]. When plant-based diets are compared with high or low extra virgin olive oil, both diets improve cardiometabolic risk profiles compared with baseline diets [261]. Decreases in LDL-C however is more pronounced after the low extra virgin olive oil diet. Dietary fruits and vegetables, abundant in the traditional Mediterranean diet have been proposed to protect against impaired endothelial function secondary to fats and oils [262].

#### Consensus of leading national nutrition and dietetic associations

Several leading dietetic associations across several countries have acknowledged that a well-planned vegan diet is healthy for everyone across the lifecycle including pregnant and lactating women [263, 264]. These countries include the United States (Academy of Nutrition and Dietetics); Canada (Dietitians of Canada); United Kingdom (British Dietetic Association and the National Health Service); Italy (Società Italiana di Nutrizione Umana); Israel (Ministry of Health); Nordic countries including Denmark, Finland, Iceland, Norway, Sweden, the Faroe Islands, Greenland and Åland (Nordic Council of Ministers); and Australia (National Health and Medical Research Council). Such information has been little publicized to the public and many health professionals.

#### Protein

Although animal protein consumption exceeds recommendations in high-, middle- and low-income countries [265], the adequacy of plant-based nutrition for elders has been queried with respect to potential reduced muscle mass and strength [266]. There is little evidence however supporting that individuals over 65 y need substantially higher protein intakes, e.g., 1 g/kg of body weight rather than the recommended 0.8 g/kg [267, 268]. Longevity experts concur that protein should be derived from plants to prevent excess activation of IGF-1, a potent growth hormone similar to insulin [269, 270]. Low levels of IGF-1 shift body’s priorities from growth and repair to maintenance and repair, thereby extending survival [271]. Although nutrition and general health are central to promoting strength in any aged cohort, exercise is a predominant means of preserving or increasing muscle strength [272, 273].

Finally, high animal protein intake has been positively associated with premature mortality (Table 1, Supplemental Materials, Animal-sourced foods and evidence supporting associated health risks), whereas high plant protein intake is inversely associated with all-cause and cardiovascular and renal disease mortality, especially among older individuals with at least one lifestyle risk factor [274–276]. Substitution of plant protein for animal protein, especially that from processed red meat, is associated with lower mortality, suggesting the importance of protein source [275].

#### Dairy

Based on historical precedent, politics, and economics, beginning with dairy surpluses as early as after World War 1, milk and dairy products remain in many national food guides [114, 115]. The nutrients from milk products can be sourced from a healthful diet of whole grains, fruits, vegetables, beans, peas, and lentils [277]. These nutrient-dense foods provide all the nutrient requirements needed by humans, without the associated health risks and harms of saturated fat and cholesterol, and lactose intolerance [278].

Once babies are weaned from the breast, they lose the ability to digest lactose. Some 68% of the world population is lactose intolerant including 36% of Americans [278]. The consumption of milk from other animals by human babies after they are weaned from breast feeding, reflects cultural evolution.

Some investigators report that ‘milk consumption does more good than harm’ [279, 280]. Such reports however do not adequately address the overall added saturated fat load from milk ingestion or consequences of intake of exogenous hormones such as IGF-1.

Thorning and colleagues, who received industry funding, concluded that the scientific evidence supports that intake of milk and dairy products contributes to meeting nutrient recommendations, and may protect against the most prevalent chronic diseases; and that few adverse effects have been reported [279, 280]. Similarly, other investigators who received industry funding have reported neutral associations between dairy products and cardiovascular and all-cause mortality [280, 281]. Curiously, despite that observation, one study’s investigators concluded that ‘it is important to investigate in more detail how dairy products can be replaced by other foods’ [281].

#### Vitamin B12

Vitamin B12 is essential for human growth and development throughout life [282]. Animal-sourced foods that contain high amounts of protein-bound vitamin B12 [283, 284] are often promoted as essential sources. However, this vitamin is produced by micro-organisms in soil and not by animals [285]. Animals including humans must obtain the vitamin directly or indirectly from other sources. In many countries, animal feed is supplemented with various ways including vitamin B12 [286]. Although not recommended for human consumption, bacteria-laden manure and unsanitary water are sources of vitamin B12 [287]. It is also found in the human intestinal tract, yet the degree to which it is absorbed in sufficient quantities and those factors that regulate its absorption, is unclear. Given our more sanitized way of life in which vegans do not readily ingest foods today with vitamin B12, they are advised to consume foods that are fortified with vitamin B12 or take a daily supplement [288, 289]. Irrespective of preferred nutrition pattern however, several factors can contribute to vitamin B12 deficiencies, e.g., long-term antacid use, alcohol abuse, smoking, gastrointestinal inflammatory conditions, and conditions that slow gut motility, as well as insufficient intake of the vitamin [290].

#### Cholesterol

The human liver makes all the cholesterol that is needed for growth and metabolism [291], thus no dietary cholesterol, predominant in animal-sourced foods, is needed in the human diet. Individuals with a cholesterol level below 3.88 mmol/L (150 mg/dL) rarely have heart disease [67]. Thus, Esselstyn argued that qualifiers typically used in reference to unhealthy foods with no nutrition value such as ‘limit’, ‘reduce’ and ‘eat in moderation’, may be misleading, as safe limits, i.e., being beneficial to heath and not harmful, have yet to be documented.

#### Fish and seafood

The consumption of fish and seafood, constituents of the Mediterranean diet, has been reported to be healthier for humans than consuming land animals [292] and generally healthy [207, 239, 293, 294]. Other evidence supports health risks comparable to the consumption of land animals [295–299]. In addition, concerns have been raised about the bio-accumulation and bio-concentration of toxic heavy metals in fish and sea food [299–301]. Today, the health concerns associated with fish and seafood consumption largely reflect the toxic effects of heavy metal bio-accumulation and bio-magnification given that fish and seafood are primary transfer mechanisms for poly-fluoroalkylated substances and several long-chain perfluoroalkyl carboxylic acids [299–305]. Since the mid-twentieth century, these synthetic chemicals have become widespread environmental contaminants. Seafood is reported to be the major dietary source contributing 38% of the estimated dietary intakes of perfluorooctanoic acid, 93% of perfluoroundecanoic acid, and 81% of perfluorooctane sulfonate [306].

Finally, DHA (docosahexaenoic acid) and EPA (eicosapentaenoic acid), which are omega-3 fatty acids that are typically found in fish, can be found in plant sources (given that plants in the form of algae are the source of these fatty acids consumed by fish), e.g., walnuts, flaxseeds, chia seeds, hemp seeds, and sea vegetables [307]. Overall, based on a review, the Physicians Committee for Responsible Medicine concluded that fish is a poor dietary choice due to its saturated fat, cholesterol, lack of fiber, as well as high levels of toxins [308].

### Constituents of the Universal Food Guide template

The range of lines of support for the healthfulness of vegan nutrition meets several of the criteria for inferring causality from associative evidence, i.e., strength of the association; consistency of findings; specificity; temporality; biological gradient (i.e., dose–response relationship); plausibility; coherence; analogy; and reversibility [309]. The strength of this association has been substantiated in a recent study with a sophisticated crossover design that compared health outcomes of a healthy omnivorous diet with those of a vegan diet in pairs of twins [187]. Within eight weeks, the vegan diet resulting in superior cardiometabolic outcome and other markers.

We sourced two evidence-based regimens for healthy vegan nutrition that met the criteria for inclusion into framing the constituents of the Universal Food Guide, i.e., maximally beneficial to health and least risk, and that could be further verified based on the extant literature. These sources are informed by some 65 years of collective global surveillance and evidence syntheses by two independent research collaboratives, founded by Michael Greger and Neal Barnard whose works are accessible through nutritionfacts.org [310] and pcrm.org (Physicians Committee for Responsible Medicine) [311], respectively. Neither has corporate or political ties, and both are non-profit. (Table 2, Translation of the science to inform the basic constituents of the proposed Universal Food Guide to serve as a template for national food guides). They provide a starting point for science-focused dialogue related to this proposed Universal Food Guide; and potentially, a new set-point for advancing the field. Distinct from the SWAD, these guidelines are consistent with being anti-oxidative and anti-inflammatory, e.g., nutrient-dense with no cholesterol and no added fat or oil; and having low glycemic load; low fatty acid composition; high macronutrient composition and quality; high micronutrient density; being alkaline; having high potassium to sodium ratio; and high fiber content (legumes, whole grains, vegetables and fruit) (Table 2). These attributes are in direct opposition to the pro-inflammatory, health-harming constituents of the SWAD [3].

**Table 2:** Translation of the science to inform the basic constituents of the proposed Universal Food Guide to serve as a template for national food guides; the dietary constituents that need to be adapted across geographic regions, and supportive evidence^a^

**Dietary constituent with serving size**^b^	**Supportive evidence**
≥ 3 servings of legumes (beans, peas, and lentils) e.g. 1–2 C cooked beans/peas/lentils; ¼ C hummus (chickpeas)	Afshin A, Micha R, Khatibzadeh S, Mozaffarian D. Consumption of nuts and legumes and risk of incident ischemic heart disease, stroke, and diabetes: A systematic review and meta-analysis. Am J Clin Nutr. 2014;100:278–88. Nanri A, Mizoue T, Takahashi Y, Kirii, K, Inoue, M, Noda M, et al. Soy product and isoflavone intakes are associated with a lower risk of type 2 diabetes in overweight Japanese women. J Nutr. 2010;140:580–586. Diet, Nutrition, and Physical Activity. A Global Perspective. A Summary of the Third Expert Report. Available at: https://www.wcrf.org/wp-content/uploads/2021/02/Summary-of-Third-Expert-Report-2018.pdf. retrieved 8 September 2024. Polak R, Phillips EM, Campbell A. Legumes: Health benefits and culinary approaches to increase intake. Clin Diabetes. 2015;33(4):198–205. Ganesan K, Xu B. Polyphenol-rich lentils and their health promoting effects. Int J Mol Sci. 2017 Nov 10;18(11):2390. 10.3390/ijms18112390. Asbaghi O, Ashtary-Larky D, Mousa A, Rezaei Kelishadi M, Moosavian SP. The effects of soy products on cardiovascular risk factors in patients with type 2 diabetes: A systematic review and meta-analysis of clinical trials. Adv Nutr. 2022;13(2):455–73. Begum N, Khan QU, Liu LG, Li W, Liu D, Haq IU. Nutritional composition, health benefits and bio-active compounds of chickpea (Cicer arietinum L.). Front Nutr. 2023 Sep 28;10:1,218,468. 10.3389/fnut.2023.1218468. Duan Y, Qi Q, Liu Z, Zhang M, Liu H. Soy consumption and serum uric acid levels: A systematic review and meta-analysis. Front Nutr. 2022 Sep 2;9:975,718. 10.3389/fnut.2022.975718. Ko KP, Park SK, Yang JJ, Ma SH, Gwack J, Shin A, et al. Intake of soy products and other foods and gastric cancer risk: a prospective study. J Epidemiol. 2013;23(5):337–43. Naureen Z, Bonetti G, Medori MC, Aquilanti B, Velluti V, Matera G, et al. Foods of the Mediterranean diet: garlic and Mediterranean legumes. J Prev Med Hyg. 2022;63(2 Suppl 3):E12-E20. Becerra-Tomás N, Díaz-López A, Rosique-Esteban N, Ros E, Buil-Cosiales P, Corella D, Estruch R, Fitó M, Serra-Majem L, Arós F, Lamuela-Raventós RM, Fiol M, Santos-Lozano JM, Díez-Espino J, et al.; PREDIMED Study Investigators. Legume consumption is inversely associated with type 2 diabetes incidence in adults: A prospective assessment from the PREDIMED study. Clin Nutr. 2018;37(3):906–13.
1 serving of berries e.g. ½ C fresh or frozen; ¼ C dried	Vahapoglu B, Erskine E, Gultekin Subasi B, Capanoglu E. Recent studies on berry bioactives and their health-promoting roles. Molecules. 2021 Dec 24;27(1):108. 10.3390/molecules27010108. Martini D, Marino M, Del Bo' C. Berries and human health: Mechanisms and evidence. Nutrients. 2023 May 29;15(11):2527. 10.3390/nu15112527. Harvard TH Chan School of Public Health. Berries are among the healthiest foods you can eat. Available at: https://www.hsph.harvard.edu/news/hsph-in-the-news/fresh-berries-are-among-the-healthiest-foods-you-can-eat/#:~:text=He%20suggested%20eating%20a%20cup,help%20promote%20a%20healthy%20gut. retrieved 8 September 2024. Hameed A, Galli M, Adamska-Patruno E, Krętowski A, Ciborowski M. Select polyphenol-rich berry consumption to defer or deter diabetes and diabetes-related complications. Nutrients. 2020 Aug 21;12(9):2538. 10.3390/nu12092538. Davicco MJ, Wittrant Y, Coxam V. Berries, their micronutrients and bone health. Curr Opin Clin Nutr Metab Care. 2016;19(6):453–57. Li R, Tao M, Xu T, Pan S, Xu X, Wu T. Small berries as health-promoting ingredients: a review on anti-aging effects and mechanisms in *Caenorhabditis elegans*. Food Funct. 2022;13(2):478–500.
3 servings of other fruits e.g. 1 medium fruit; ¼ dried fruit	Dreher ML. Whole fruits and fruit fiber Emerging health effects. Nutrients. 2018 Nov 28;10(12):1833. 10.3390/nu10121833. Slavin JL, Lloyd B. Health benefits of fruits and vegetables. Adv Nutr. 2012;3(4):506–16. Du H, Li L, Bennett D, Yang L, Guo Y, Key TJ, et al.; China Kadoorie Biobank study. Fresh fruit consumption and all-cause and cause-specific mortality: findings from the China Kadoorie Biobank. Int J Epidemiol. 2017;46(5):1444–55. Bayili RG, Abdoul-Latif F, Kone OH, Diao M, Bassole IHN, Dicko MH. Phenolic compounds and antioxidant activities in some fruits and vegetables from Burkina Faso. Afr. J. Biotechnol. 2011;10:13,543–47.
1 serving of cruciferous vegetables e.g. ½ C chopped; 1 T horseradish	Ağagündüz D, Şahin TÖ, Yılmaz B, Ekenci KD, Duyar Özer Ş, Capasso R. Cruciferous vegetables and their bioactive metabolites: from prevention to novel therapies of colorectal cancer. Evid Based Complement Alternat Med. 2022 Apr 11;2022:1,534,083. 10.1155/2022/1534083. Blekkenhorst LC, Sim M, Bondonno CP, Bondonno NP, Ward NC, Prince RL, et al. Cardiovascular health benefits of specific vegetable types: A narrative review. Nutrients. 2018 May 11;10(5):595. 10.3390/nu10050595. Blekkenhorst LC, Bononno CP, Lewis JR, Devine A, Zhu K, Lim WH, et al. Cruciferous and allium vegetable intakes are inversely associated with 15-year atherosclerotic vascular disease deaths in older adult women. J Am Heart Assoc. 2017 Oct 24;6(10):e006558. 10.1161/JAHA.117.006558.
2 servings of greens e.g. 1 C raw; ½ C cooked	Blekkenhorst LC, Sim M, Bondonno CP, Bondonno NP, Ward NC, Prince RL, et al. Cardiovascular health benefits of specific vegetable types: A narrative review. Nutrients. 2018 May 11;10(5):595. 10.3390/nu10050595. Mohammed SG, Qoronfleh MW. Vegetables. Adv Neurobiol. 2020;24:225–277. 10.1007/978-3-030-30402-7_9. Wallace TC, Bailey RL, Blumberg JB, Burton-Freeman B, Chen CO, Crowe-White KM, et al. Fruits, vegetables, and health: A comprehensive narrative, umbrella review of the science and recommendations for enhanced public policy to improve intake. Crit Rev Food Sci Nutr. 2020;60(13):2174–211. Crous-Bou M, Molinuevo JL, Sala-Vila A. Plant-rich dietary patterns, plant foods and nutrients, and telomere length. Adv Nutr. 2019;10(Suppl_4):S296-S303.
2 servings of other vegetables e.g. ½ C non-leafy vegetables	Blumfield M, Mayr H, De Vlieger N, Abbott K, Starck C, Fayet-Moore F, et al. Should we 'Eat a Rainbow'? An umbrella review of the health effects of colorful bioactive pigments in fruits and vegetables. Molecules. 2022 Jun 24;27(13):4061. 10.3390/molecules27134061. Renna M, Signore A, Paradiso VM, Santamaria P. Faba greens, globe artichoke's offshoots, crenate broomrape and summer squash greens: Unconventional vegetables of Puglia (Southern Italy) with good quality traits. Front Plant Sci. 2018 Mar 27;9:378. 10.3389/fpls.2018.00378. Ahmed T, Wang CK. Black garlic and Its bioactive compounds on human health diseases: A review. Molecules. 2021 Aug 19;26(16):5028. 10.3390/molecules26165028. Farhat Z, Scheving T, Aga DS, Hershberger PA, Freudenheim JL, Hageman Blair R, et al. Antioxidant and antiproliferative activities of several garlic forms. Nutrients. 2023 Sep 22;15(19):4099. 10.3390/nu15194099. Mohd Sahardi NFN, Makpol S. Suppression of Inflamm-aging by *Moringa oleifera* and *Zingiber officinale* Roscoe in the prevention of degenerative diseases: A review of current evidence. Molecules. 2023 Aug 3;28(15):5867. 10.3390/molecules28155867. Mao QQ, Xu XY, Cao SY, Gan RY, Corke H, Beta T, et al. Bioactive compounds and bioactivities of ginger (*Zingiber officinale* Roscoe). Foods. 2019 May 30;8(6):185. 10.3390/foods8060185. Kaur Ch., Kapoor H.C. Antioxidants in fruits and vegetables the millennium’s health. Int. J. Food Sci. Technol. 2001;36:703–25. Igbokwe G.E., Aniakor G.C., Anagonye C.O. Determination of β-carotene & vitamin C content of fresh green pepper (*Capsicum annnum*), fresh red pepper (*Capsicum annum*) and fresh tomatoes (*Solanum lycopersicum*) Fruits. Bioscientist*. *2013;1:89–93. Collins EJ, Bowyer C, Tsouza A, Chopra M. Tomatoes: An extensive review of the associated health impacts of tomatoes and factors that can affect their cultivation. Biology (Basel). 2022 Feb 4;11(2):239. 10.3390/biology11020239. Palomo I, Fuentes E, Padró T, Badimon L. Platelets and atherogenesis: Platelet anti-aggregation activity and endothelial protection from tomatoes (Solanum lycopersicum L.). Exp Ther Med. 2012;3(4):577–84. Mohanraj R, Sivasankar S. Sweet potato (Ipomoea batatas [L.] Lam)–a valuable medicinal food: a review. J Med Food. 2014;17(7):733–41. McLean RM, Wang NX. Potassium. Adv Food Nutr Res. 2021;96:89–121. Wang S, Nie S, Zhu F. Chemical constituents and health effects of sweet potato. Food Res Int. 2016;89(Pt 1):90–116. Asemani Y, Zamani N, Bayat M, Amirghofran Z. Allium vegetables for possible future of cancer treatment. Phytother Res. 2019;33(12):3019–39. Czech A, Szmigielski M, Sembratowicz I. Nutritional value and antioxidant capacity of organic and conventional vegetables of the genus Allium. Sci Rep. 2022 Nov 4;12(1):18,713. 10.1038/s41598-022-23497-y. Nicastro HL, Ross SA, Milner JA. Garlic and onions: their cancer prevention properties. Cancer Prev Res (Phila). 2015;8(3):181–9. Mandrich L, Esposito AV, Costa S, Caputo E. Chemical composition, functional and anticancer properties of carrot. Molecules. 2023 Oct 19;28(20):7161. 10.3390/molecules28207161.
3 servings of whole grains e.g. ½ C hot cereal; 1 slice bread	Ma X, Tang WG, Yang Y, Zhang QL, Zheng JL, Xiang YB. Association between whole grain intake and all-cause mortality: a meta-analysis of cohort studies. Oncotarget. 2016;7(38):61,996–2005. Khan J, Khan MZ, Ma Y, Meng Y, Mushtaq A, Shen Q, et al. Overview of the composition of whole grains' phenolic acids and dietary fibre and their effect on chronic non-communicable diseases. Int J Environ Res Pub Health. 2022 Mar 5;19(5):3042. 10.3390/ijerph19053042. Călinoiu LF, Vodnar DC. Whole grains and phenolic acids: A Review on bioactivity, functionality, health benefits and bioavailability. Nutrients. 2018 Nov 1;10(11):1615. https://doi.org/10.339/nu10111615. Wei X, Yang W, Wang J, Zhang Y, Wang Y, Long Y, et al. Health effects of whole grains: A bibliometric analysis. Foods. 2022 Dec 18;11(24):4094. https://doi.org/10.3390/foods11244094 Capurso C. Whole-grain intake in the Mediterranean diet and a low protein to carbohydrates ratio can help to reduce mortality from cardiovascular disease, slow down the progression of aging, and to improve lifespan: A review. Nutrients. 2021 Jul 25;13(8):2540. 10.3390/nu13082540. Develaraja S, Reddy A, Yadav M, Jain S, Yadav H. Whole grains in amelioration of metabolic derangements. J Nutrit Health Food Sci. 2016;4(4):1–11 Wu X, Guo T, Luo F, Lin Q. Brown rice: a missing nutrient-rich health food. Food Sci Hum Wellness. 2023;12(5):1458–70. Saleh ASM, Wang P, Wang N, Yang L, Xiao Z. Brown rice versus white rice: Nutritional quality, potential health benefits, development of food products, and preservation technologies. Compr Rev Food Sci Food Saf. 2019;18(4):1070–96.
1 serving of ground flaxseeds e.g. 1 tbsp ground Flaxseeds are high in fiber, omega-3 fatty acids, and phytochemicals called lignans; and also found in chia seeds and walnuts One tablespoon (7 g) contains 2 g of polyunsaturated fatty acids (includes the omega 3s), 2 g of dietary fiber and 37 cal^c^ See other sources of omega 3s below, under Additional Notes	van den Driessche JJ, Plat J, Mensink RP. Effects of superfoods on risk factors of metabolic syndrome: a systematic review of human intervention trials. Food Funct. 2018;9(4):1944–66. Guo S, Ge Y, Na Jom K. A review of phytochemistry, metabolite changes, and medicinal uses of the common sunflower seed and sprouts (Helianthus annuus L.) Chemistry Central Journal. 2017;11(1):95. Guo S, Ge Y, Na Jom K. A review of phytochemistry, metabolite changes, and medicinal uses of the common sunflower seed and sprouts (Helianthus annuus L.) Chemistry Central Journal. 2017;11(1):95. https://10.1186/s13065-017-0328-7. Barsby JP, Cowley JM, Leemaqz SY, Grieger JA, McKeating DR, Perkins AV, et al. Nutritional properties of selected superfood extracts and their potential health benefits. Peer J. 2021 Nov 26;9:e12525. 10.7717/peerj.12525. Parikh M, Maddaford TG, Austria JA, Aliani M, Netticadan T, Pierce GN. Dietary flaxseed as a strategy for improving human health. Nutrients. 2019 May 25;11(5):1171. 10.3390/nu11051171. Marcinek K, Krejpcio Z. Chia seeds (Salvia hispanica): health promoting properties and therapeutic applications – a review. Rocz Panstw Zakl Hig. 2017;68(2):123–29. Kris-Etherton PM. Walnuts decrease risk of cardiovascular disease: a summary of efficacy and biologic mechanisms. J Nutr. 2014;144(4 Suppl):547S-54S. Physicians Committee for Responsible Medicine. Omega-3 fatty acids and plant-based diets. Available at: https://www.pcrm.org/good-nutrition/nutrition-information/omega-3. retrieved 8 September 2024.
1 serving of nuts and seeds e.g. ¼ C nuts; 2 tbsp nut butter	De Souza RGM, Schincaglia RM, Pimentel GD, Mota JF. Nuts and human health outcomes: a systematic review. Nutrients. 2017;9(12):1311. 10.3390/nu9121311. Kim Y, Keogh J, Clifton PM. Nuts and cardio-metabolic disease: a review of meta-analyses. Nutrients. 2018;10(12):1935. https://doi.org/10.3390/nu10121935 Coates AM, Hill AM, Tan SY. Nuts and cardiovascular disease prevention. Curr Atheroscler Rep. 2018;20(10):48. 10.1007/s11883-018-0749-3. Tan S-Y, Georgousopoulou EN, Cardoso BR, Daly RM, George ES. Associations between nut intake, cognitive function and non-alcoholic fatty liver disease (NAFLD) in older adults in the United States: NHANES 2011–14. BMC Geriatrics. 2021;21(1):1–12. Cardoso BR, Tan S-Y, Daly RM, Dalla Via J, Georgousopoulou EN, George ES. Intake of nuts and seeds is associated with a lower prevalence of nonalcoholic fatty liver disease in US adults: findings from 2005–2018 NHANES. J Nutr. 2021;151(11):3507–15. Afshin A, Micha R, Khatibzadeh S, Mozaffarian D. Consumption of nuts and legumes and risk of incident ischemic heart disease, stroke, and diabetes: A systematic review and meta-analysis. Am J Clin Nutr. 2014;100:278–88. de Souza RGM, Schincaglia RM, Pimentel GD, Mota JF. Nuts and human health outcomes: A systematic review. Nutrients. 2017 Dec 2;9(12):1311. 10.3390/nu9121311.
Herbs and spices (for nutrients and to flavor food, rather than fat, sugar, and salt and other additives)	Xu XY, Meng X, Li S, Gan RY, Li Y, Li HB. Bioactivity, health benefits, and related molecular mechanisms of curcumin: Current progress, challenges, and perspectives. Nutrients. 2018 Oct 19;10(10):1553. 10.3390/nu10101553. Gajewska D, Kęszycka PK, Sandzewicz M, Kozłowski P, Myszkowska-Ryciak J. Intake of dietary salicylates from herbs and spices among adult Polish omnivores and vegans. Nutrients. 2020 Sep 6;12(9):2727. https://doi.org/10.3390/nu12092727 Mackonochie M, Rodriguez-Mateos A, Mills S, Rolfe V. A scoping review of the clinical evidence for the health benefits of culinary doses of herbs and spices for the prevention and treatment of metabolic syndrome. Nutrients. 2023 Nov 22;15(23):4867. 10.3390/nu15234867. Isbill J, Kandiah J, Kružliaková N. Opportunities for health promotion: Highlighting herbs and spices to improve immune support and well-being. Integr Med (Encinitas). 2020;19(5):30–42. Vázquez-Fresno R, Rosana ARR, Sajed T, Onookome-Okome T, Wishart NA, Wishart DS. Herbs and spices—Biomarkers of intake based on human intervention studies—A systematic review. Genes Nutr. 2019 May 22;14:18. 10.1186/s12263-019-0636-8. Jiang TA. Health benefits of culinary herbs and spices. J AOAC Int. 2019;102(2):395–411. Balasubramanian S, Roselin P, Singh KK, Zachariah J, Saxena SN. Postharvest processing and benefits of black pepper, coriander, cinnamon, fenugreek, and turmeric Spices. Crit Rev Food Sci Nutr. 2016;56(10):1585–607. Hewlings SJ, Kalman DS. Curcumin: A review of its effects on human health. Foods. 2017 Oct 22;6(10):92. 10.3390/foods6100092. Dludla PV, Cirilli I, Marcheggiani F, Silvestri S, Orlando P, Muvhulawa N, et al. Bioactive properties, bioavailability profiles, and clinical evidence of the potential benefits of black pepper (*Piper nigrum*) and red pepper (*Capsicum annum*) against diverse metabolic complications. Molecules. 2023 Sep 11;28(18):6569. 10.3390/molecules28186569.
Beverages e.g. water, green tea, hibiscus tea	Unno K, Nakamura Y. Green tea suppresses brain aging. Molecules. 2021 Aug 12;26(16):4897. 10.3390/molecules26164897. Tang GY, Meng X, Gan RY, Zhao CN, Liu Q, Feng YB, et al. Health functions and related molecular mechanisms of tea components: An update review. Int J Mol Sci. 2019 Dec 8;20(24):6196. https://doi.org/10.3390/ijms20246196. Mokra D, Joskova M, Mokry J. Therapeutic effects of green tea polyphenol (‒)-Epigallocatechin-3-Gallate (EGCG) in relation to molecular pathways controlling inflammation, oxidative stress, and apoptosis. Int J Mol Sci. 2022 Dec 25;24(1):340. 10.3390/ijms24010340. Ellis LR, Zulfiqar S, Holmes M, Marshall L, Dye L, Boesch C. A systematic review and meta-analysis of the effects of Hibiscus sabdariffa on blood pressure and cardiometabolic markers. Nutr Rev. 2022;80(6):1723–37. Montalvo-González E, Villagrán Z, González-Torres S, Iñiguez-Muñoz LE, Isiordia-Espinoza MA, Ruvalcaba-Gómez JM, et al. Physiological effects and human health benefits of Hibiscus sabdariffa: A Review of Clinical Trials. Pharmaceuticals (Basel). 2022 Apr 12;15(4):464. 10.3390/ph15040464.
Physical Activity^d^	
Physical activity/exercise e.g. 90 min moderately-intense (can talk but falteringly) or 40 min vigorously-intense (unable to speak continuously)	Malhotra A, Noakes T, Phinney S. It is time to bust the myth of physical inactivity and obesity: you cannot outrun a bad diet. Br J Sports Med. 2015;49(15):967–8.

^a^Sources: Greger M. How Not to Die. [Evidence Synthesis Monograph]. Flatiron Books:New York, NY, 2015; Greger M. How Not to Diet. [Evidence Synthesis Monograph]. Flatiron Books:New York, NY, 2019; Physicians Committee for Responsible Medicine. Good Nutrition. Available at: https://www.pcrm.org and Power Plate: A Model for Plant-Based Healthy Eating from PCRM available at: https://yummyplants.com/vegan-nutrition/power-plate-a-new-model-for-healthy-eating-from-pcrm/#:~:text=Its%20recommended%20dietary%20staples%20are,hypertension%2C%20and%20type%202%20diabetes. retrieved 8 September 2024

^b^Serving sizes are a guide given individuals differ by age, sex, and metabolic needs. Proportionality of serving sizes across constituent categories is recommended

^c^Source: https://www.mayoclinic.org/healthy-lifestyle/nutrition-and-healthy-eating/expert-answers/flaxseed/faq-20058354#:~:text=Flaxseed's%20health%20benefits%20come%20from,dietary%20fiber%20and%2037%20calories. retrieved 8 September 2024

^d^Physical activity is typically encouraged depending on the individual’s physical status and health condition

Additional Notes on Folate, Iodine, Omega 3 fatty acids, and Vitamin B12:

Folate: Many fruits and vegetables are good sources of folate. In some countries, bread, cereal, flour, cornmeal, pasta, rice, and other grain products are fortified with folic acid. For plant-based sources of folate see: National Institute for Health. Folate. Fact Sheet for Health Professionals. Available at: https://ods.od.nih.gov/factsheets/Folate-HealthProfessional/#:~:text=Folate%20is%20naturally%20present%20in,)%20%5B4%2C12%5D. retrieved 8 September 2024

Iodine: An essential element for human health. Adults require 150 mcg (μg) daily (National Institute of Health, How Much Iodine Do I Need. Available at: https://ods.od.nih.gov/factsheets/Iodine-Consumer/). It is a natural constituent of sea vegetables (sea weeds) which are not always commonly available. High-income countries add iodine to table salt. Although table salt in homes contributes to high sodium intake, many individuals get more sodium from processed and ultra-processed foods which has resulted in excessive sodium consumption, in turn, hypertension and related health problems. Thus, when ingested through ultra-processed foods, sodium intake can be high even if the food does not taste salty. Regulatory bodies and governments need to consider ways that certain foods can be enriched with iodine, other than salt. Refer to other detailed sources for recommended daily amounts for children, pregnant and lactating women, and elders

Omega 3s: There are three main types of omega-3 fatty acids. Eicosapentaenoic acid (EPA): found in fish and their oils (mackerel, cod, sardines, salmon); Docosahexaenoic acid (DHA): also in fish; and Alpha-linolenic acid (ALA): in plant foods (beans, canola oil, flaxseeds, chia seeds, walnuts, hemp seeds, and their oils. These foods are much richer sources of ALA and can contribute to meeting omega-3 intake goals of 1.1 to 1.6 g daily for adults, depending on age and sex). Available at: https://www.webmd.com/healthy-aging/omega-3-fatty-acids-fact-sheet; and https://ods.od.nih.gov/factsheets/Omega3FattyAcids-HealthProfessional/. both retrieved 8 September 2024). Omega 3 s may need to be supplemented in those consuming plant-based diets

Vitamin B12: An essential vitamin for growth, repair, and maintenance. Nutritional yeast fortified with vitamin B12 is one source or it can be taken as a supplement. Governments need to consider fortifying appropriate foods with vitamin B12. As a supplement, at least 2000 mcg (μg) cyanocobalamin for adults once a week (or at least 50 mcg daily) ideally as a chewable sublingual, or liquid supplements taken on an empty stomach. Because of our sanitized way of life, we are less likely to access vitamin B12 through drinking water and vegetable sources (Pawlak R, Parrott SJ, Raj S, et al. How prevalent is vitamin B12 deficiency among vegetarians? Nutr Rev. 2013;71(2):110–7; Watanabe F, Yabuta Y, Bito T, Teng F. Vitamin B12-containing plant food sources for vegetarians. Nutrients 2014; 6:1861–73). Refer to other detailed sources for recommended daily amounts for children, pregnant and lactating women, and elders

Both nutrition guidelines [310, 311] provide all of an average adult’s daily macro- and micro-nutrient needs including substantial fiber, protein, iron, calcium, zinc, and B vitamins with the exception of vitamin B12 which needs to be supplemented [312]; examples of serving sizes across food groups are shown in Table 2. Portions or serving sizes are established proportionally across food categories in order to adjust for individual body sizes, metabolic demands, and gender differences. Greger’s food constituents and recommendations include daily [310]:

- At least 3 servings of legumes including beans, peas, lentils, soybeans (or soy nuts), and peanuts;
- At least 4 servings of berries and other fruit;
- At least 5 servings of cruciferous vegetables, greens, and other vegetables;
- And at least 3 servings of whole grains.

In addition, these recommendations include one serving daily each of flaxseeds; nuts and seeds; and herbs and spices. Plant-based sources of omega 3 fatty acids include flaxseeds, nuts such as walnuts, and seeds, in addition [313]. These sources need to be assured to achieve optimal levels without supplementation, or with supplementation or consuming omega 3 enriched foods. Exercise is categorized as an additional recommendation given its importance to overall health and wellbeing.

Barnard’s four food groups [311], concur qualitatively with Greger’s recommendations, to be consumed daily are: at least 3 servings of fruit; at least 2 servings of legumes; at least 5 servings of whole grains; and at least 4 servings of vegetables. These are illustrated proportionally in the Power Plate with examples [311]. The number of servings in both nutrition guidelines is variable, being dependent on the size and needs of a given individual.

Neither nutrition guideline includes animal-sourced foods nor added sugar or salt; and no UPF. They acknowledge iodine is essential and typically added to table salt, but can be obtained through plant sources. Both agree, that although protein from fish has constituted part of the traditional Mediterranean diet, bio-concentration of toxic pollutants in the flesh of fish and other seafood, makes it a less healthy choice compared with a whole food, low fat, vegan diet.

The proposed Universal Food Guide aims to use no ambiguous, qualifying terminology in relation to unhealthy foods including sugar; fats and oils; refined foods; UPF; animal products such as meat, dairy; eggs; poultry; and fish and seafood.

National food guides based on the Universal Food Guide template need to be accompanied by information addressing commonly-perpetuated nutrition myths. Such myths include: humans are natural carnivores; children need to drink milk after weaning; children and elders might not have sufficient nutrition with vegan nutrition, e.g., protein and calcium; all foods can be eaten ‘in moderation’; vegan food is expensive; and athletes need meat [185, 227, 228, 314–321]. These myths detract from people making healthy food choices. They are often promulgated through corporate marketing mis-information by those with vested interests; and potentially by well-intentioned food guide advisory committee members, believing that a food guide will be more acceptable to the public if meat, dairy, and eggs, and health-harming products are promoted, albeit in moderation.

Despite the evidence supporting the healthfulness of a whole-food, low-fat vegan eating pattern, some critics have challenged the notion of its superiority [185, 186]. When examining eating patterns, the critical question reasonably becomes to what extent do their health outcomes surpass the health benefits of a whole-food, low-fat, vegan eating pattern, in the short- and long-terms?

### Implications

#### Population health

According to the WHO, ‘*Health is a fundamental human right*’ [322]. The WHO further acknowledges that ‘*The achievement of any State in the promotion and protection of health is of value to all*’ [323]. Despite recent critique [5], the publication of the 2019 Report of EAT Lancet Commission was seminal in increasing global awareness about the increasingly pervasive SWAD, its health-harming consequences, and the need to return to more whole-food, sustainable, plant-based eating patterns [1].

The public needs to be aware that no safe limits have been documented for the consumption of meat, poultry, dairy, and eggs, as well as alcohol. And, further, there are no safe limits for synthetic color dyes and other additives commonly used in UPF, e.g., sunset yellow fcf, allure red, tartrazine, and titanium dioxide which are permitted in most countries other than European countries [324].

That healthy whole-food, low-fat vegan nutrition is difficult to adhere to [231] and is expensive are myths which warrant being dispelled in the eyes of the public as well as health professionals. Plant-based diets are less expensive than standard omnivorous alternatives as well as healthier [325–328]. Furthermore, healthy plant-based diets reduce health care costs [329–332]. One econometric analysis conducted by the Office of Health Economics in the United Kingdom showed that adoption of plant-based eating patterns could save the National Health Service about 6.7 billion pounds annually (i.e., over $9 billion US) [333]. The study concluded that public health decision makers have a responsibility to institute strategies that support the public in transitioning from the SWAD to evidence-based plant-based nutrition including vegan diets.

Given the engineered influence of the ‘commercial determinants of health’ on the public’s food choices, changing the social and political environments that are driving food systems and food guides, needs to be the primary focus of public health authorities [334, 335] combined with public nutrition education programs. Societal environments that facilitate, rather than impede adoption of healthy eating patterns such as in the ‘food deserts’ in some American cities, and support adherence to healthy eating, are needed to ensure that the healthy choice is the easy choice [49, 168, 336].

Subsidized meat, dairy, and eggs artificially reduce their market costs to the public [337]. Although the DGA recommend that half of an adult’s dietary intake should be fruits and vegetables, only a fraction of a percent of government subsidies go towards these nutrient-dense foods. The majority goes towards meat and dairy products, whose consumption is linked to poor public health outcomes.

Population-based interventions that regulate food ingredients such as salt at time of production, the principal source in high-income countries, have considerable potential to improve public health outcomes. Salt added in cooking and at the table however, is often the dominant source in countries with developing economies, thus interventions based on education and behavior change can be particularly effective in these contexts [158, 337]. Across income levels, countries can benefit from the integration of population-based public health campaigns and individual behavioral change interventions [338].

Disease-oriented organizations such as those for heart disease, stroke, diabetes, and osteoporosis typically provide dietary guidelines to reduce the risk of these diseases. Such organizations however are not unbiased as they are often subsidized by ‘big food’ industries, which is reflected in their dietary recommendations [339]. When the outcomes of the eating pattern recommended by the American Heart Association, for example, were compared with the cardiometabolic outcomes of a healthy vegan diet [225], C-reactive protein, a risk marker of adverse outcomes, was significantly lower in those who had a plant-based eating pattern. A recent systematic review concluded greater adherence to a whole-food, plant-based eating pattern, reduces risks of chronic NCDs, diseases that these disease-organizations represent [340]. Thus, plant-based nutrition warrants being promoted foremost by such disease-related organizations.

Despite the power of healthy nutrition in supporting optimal health, medical schools devote minimal hours to nutrition in their curricula [18, 341]. Initiatives such as Klaper’s ‘Moving Medicine Forward’ is helping to address this deficiency in the United States [342].

With respect to the most recent assault on global health, the COVID-19 pandemic warrants mention. At pandemic onset, there were calls to ramp up addressing NCD risk factors including nutrition, to reduce SARS-CoV-2 infection or, if infected, reduce the severity of COVID-19 [343–345]. These calls for action were based on multi-morbidity data from northern Italy that reported the presence of NCDs was directly related to risk of COVID-19 deaths. Shortly after the release of those data, diet characterized by healthy plant-based foods was reported to be associated with lower risk and severity of COVID-19 [346].

#### Regulation and governance

World bodies have long called for population-level interventions to align nutrition recommendations with the extant evidence [42, 347–350]. Even within public institutions such as schools and hospitals, healthy plant-based food has been less accessible than health-harming food [351, 352].

As food systems have become increasingly commercially/politically-driven rather than evidence-driven, revising corporate law has been proposed [353]. The mandate of corporations is to generate profits for their shareholders rather than serve as a vehicle for human rights, social justice, and environmental sustainability [354]. Corporate businesses are structured to keep costs low including staff wages along with sub-optimal benefits and working conditions, and producing cheap, addictive, poor quality products such as UPF. To address the discordance between the roles of government and corporations with respect to public health and public health policy, Wiist and Hinkley [354] have called for banning corporate political activity; strengthening government regulatory agencies; enforcing corporate adherence to the United Nations Compact on Human Rights; and revising corporate law. Robert Hinkley, an American corporate lawyer, proposed a Code of Corporate Citizenship that states ‘The duty of directors (of corporations) henceforth shall be to make money for shareholders *but not at the expense of the environment, human rights, public health and safety, dignity of employees, and the welfare of the communities in which the company operates.’* [355]. Hinkley’s position is not only ethically defensible but also supports the notion that ‘*Governments should govern, and corporations should follow the rules*’ [356].

Despite urgent calls to world bodies for definitive action, the WHO withdrew its support in 2019 from an initiative promoting a global move towards plant-based food and nutrition [357]. This was based solely on Italian ambassador Gian Lorenzo Cornado’s ‘objection to the science’. Further, Cornado argued that the move could lead to the loss of millions of jobs in ‘animal husbandry’ and in the production of ‘unhealthy’ foods. Rather than these arguments being viewed as support for farming diversification and shift to crops and whole foods that humans can consume to support their health, retaining unsustainable animal husbandry and other health-harming food industries were prioritized to largely support corporate interests.

Global cooperation is essential to regulate tactics used by industry that normalize the consumption of animal-sourced foods and UPF [60, 61, 161, 358]. Such tactics include industry interference with the legislative process; using front groups to act on industry behalf; questioning the evidence of substance harm and the effectiveness of harm-reducing interventions which are designed to confuse the public; and appearing responsible in the eyes of the public, journalists, and policy makers [359–362]. An additional tactic is the highly profitable ‘super-marketization’ that is targeted disproportionately toward vulnerable groups (children and minority groups) which has created health inequities and cultural shifts, and a barrier to adhering to healthy nutritional guidelines [168, 363–365].

The evidence supporting animal-sourced foods and UPF being injurious to human health is downplayed in the DGA [189]. For example, the International Agency for Research on Cancer, an independent body responsible for rating the cancer risk of products and foods [366], classifies red meat as a class 2 carcinogen; and processed meats as class 1 carcinogens comparable to tobacco, asbestos, and plutonium [366]. Despite these classifications, these health-harming products continue to appear in food guides. Similarly, with the *proviso* to consume these carcinogenic foods ‘in moderation’, UPF also contains multiple carcinogens [66, 153, 335]. The full extent of the long-term consumption of UPF remains to be fully realized.

Food guide advisory committee members have been reported to have substantial conflicts of interest with industries such as ‘big food’ and ‘big pharma’ [171], or political affiliations whereby they profit directly from the recommendations of publicly-funded food guides [75, 367–369]. Academics whose work is supported by corporations also have a commercial bias in their research and research reporting [368–371]. Individuals with these conflicts of interest necessarily need to be excluded from serving on national food guide advisory committees.

In adopting the Universal Food Guide template, the responsibility of advisory committee members (with no conflicts of interest) in developing national food guides, is to establish within its food categories, how to best to maximize the public’s health within their unique contexts and to minimize harm, without contravening the science. Given their implicit knowledge of locally and regionally available and accessible whole foods, their role is to identify foods that are nutritionally-equivalent to those in the Universal Food Guide; ensure that their overall proportions and/or relative serving size units are understood and are appropriate for that country; and advise on cooking methods that are healthy and indigenous to that country.

Criticisms of food guides in industrialized countries with diverse populations, which likely reflect concerns in many countries, is the lack of attention to factors such as socioeconomic, geographic, language, culture, religion, values, and food availability, and food insecurity issues [372, 373]. Food guide advisory committee members’ primary goal is to address these issues including working with their governments and health policy authorities, so the evidence-informed principles of the Universal Good Guide can most effectively be aligned across cultures and regions. Countries such as Thailand with a long-standing Buddhist tradition could have an advantage in advancing vegan nutrition in that context.

Over recent decades, the inertia of public institutions in translating and implementing knowledge in the public’s interest due to conflicts of interest has contributed to the erosion of public trust [374]. To regain this trust in institutions related to food, Garza and colleagues have argued that food science transparency needs to be the norm [172].

Although corporations would like to deflect responsibility to individuals, the odds are stacked against the public due to a system that has long favored corporate interests over public interests [172, 375]. In turn, governments collude by holding the public accountable for their policies [334].

Finally, over the past 60 years, the nutrient quality and density of fruits and vegetables has deteriorated. Yields, appearance, and transportability have taken precedent over the nutrient quality of fruits and vegetables which is of no interest to agribusinesses [376, 377]. Over that time, the 70 most common fruits and vegetables have undergone an average loss 16% of calcium, 27% loss of vitamin C, and over 50% reduction in iron levels. Tomato production has incurred the greatest loss (25% loss in calcium, and over 50% of its vitamin) [376]. When yields go up, nutrient density goes down. Regulators, ostensibly committed to the public, have a responsibility to regulate agribusinesses to ensure that food products are developed for maximal nutritional value in addition to other qualities such as size, color, appearance, and transportability.

#### Environment, climate and planetary health

The Universal Food Guide recommendations align with the United Nations sustainable development goals [72, 378, 379]. Adverse shifts in eating patterns and agricultural practices [380] have had devastating effects on planetary health, as has UPF [70, 381, 382]. These shifts have impacted planetary health through their effects on climate including air pollution, soil degradation, water pollution, and deforestation [383–386]. The production of plant-based foods such as fruits and vegetables, whole grains, beans, peas, nuts, and lentils, uses less energy, land, and water, and produces less greenhouse gas emissions and maximizes absorption of greenhouse gases, than the production of animal-sourced foods. The United Nations and others have called for a ban on industrial animal agriculture given its substantial contributions to global greenhouse gas emissions [72–74, 387].

The environmental impacts of various eating patterns have been compared by Scarborough and colleagues [388]. Individuals consuming the most animal-sourced foods had the greatest negative environmental impact with vegans demonstrating the least impact, followed by vegetarians, fish-eaters, and finally meat-eaters. The investigators concluded that animal-based food consumption should be reduced.

Although largely neglected by the Intergovernmental Panel on Climate Change [389], the livestock sector contributes more greenhouse gas emissions than the transportation sector, for example, according to a landmark 2006 FAO report [383]. The proportion of greenhouse emissions from factory farming ranges up to 51% [383]. Goodland and Anhang [71] conducted a detailed analysis of the contribution of factory farming to greenhouse gas emissions. The report included ‘uncounted, overlooked and misallocated’ livestock-related greenhouse gas emissions which, when included, totaled over 51%. Recently, to more accurately assess the contribution of green gas emissions from animal agriculture, Rao conducted a global sensitivity analysis and concluded that animal agriculture is the leading cause of climate change, responsible for 87% of greenhouse gas emissions [390].

Global policy action is needed to reduce global temperatures with a healthy sustainable eating pattern [59, 71, 72, 379, 391–401] such as that reflected in the Universal Food Guide template. Reducing meat consumption in wealthy nations, agricultural land could be rewilded and a further 100 gigatonnes of carbon dioxide equivalent sequestered [402]. Currently, a large percentage of grains grown in the United States is used for animal feed rather than food for people, with over 70% of soy and 36% of corn produced in the United States being consumed by livestock [385]. Simply put, factory farming is poor economics.

Finally, in addition to hormone administration in factory farming [403], 80% of all antibiotics are used in factory farming in the United States (68% globally), necessitated because of the unsanitary crowded housing conditions for animals and high risk of disease [404, 405]. These hormones and antibiotics are ingested by people when they consume animal-sourced foods. The consumption of hormones changes children’s growth patterns and the consumption of antibiotics, contributes to antibiotic resistant bacteria [406–408] which has been described as a global crisis [409]. This effect is potentially amplified given the widespread detection of these drugs in all forms of water supplies [410].

#### Ethics

Three levels of ethical considerations are associated with the ubiquitous omnivorous SWAD. First, health professionals have been called upon to practice according to ethical ‘best’ practice standards, as a moral imperative [58, 411, 412] in general, and should counsel patients in plant-based nutrition [412].

Second is the question of whether factory farming is morally justified. Today, for most people, consuming animals and their products is a personal choice based on their learning and social and cultural conditioning influenced heavily by external influences, rather than a health necessity [63–65, 116]. Over 80 billion land animals and trillions of sea animals are estimated to be sacrificed annually [383, 413]. Inherent in factory farming is systemic abuse of sentient animals which includes sea animals [414–417]. Concentrated farming operations involve unnatural confinement of animals living in their excrement. Debeaking, teeth extraction, and tail cropping are common practices given animals will turn on each other when stressed and suffering [116]. In addition to other farming practices that most people would find abhorrent and distressing, these practices are intentionally kept out of public view. For example, Ag-Gag laws in the United States and other countries prohibit transparency regarding factory farming practices [418]. Corporations strive to remove association of the end products of factory farming that appear in supermarkets and grocery stores, with slaughtering processes [419, 420].

Finally, there is the under acknowledged human cost of the operation of slaughterhouses. Traumatic stress syndrome for workers who slaughter for a living whether on the killing floor, macerate live male chicks, or operate CO_2_ gas chambers to suffocate animals, has been documented [421–423]. Factory farming practices and their cost to the workers who make a living in those industries and to the environment, warrant being more transparent.

## Summary and conclusions

Adoption of the principles of the Universal Food Guide by countries globally as a template for developing their national food guides, has the capacity to reduce the prevalence of nutrition-related NCDs; eliminate related human suffering and premature death; and reduce exorbitant, unsustainable illness care costs; as well as contribute to a healthier environment and planet, and animal welfare. The findings of our integrative review support the conclusion of the 2019 EAT Lancet Commission report on healthy diets from sustainable food systems, specifically, *“Food is the single strongest lever to optimize human health and environmental sustainability on Earth.”*

## Supplementary Information

Supplementary Material 1.

## Data Availability

No databases were generated or analyzed for this study.

## Abbreviations

CLGSI: Chronic low-grade systemic inflammation
CSPI: Center for Science in the Public Interest
GBD: Global burden of diseases
NCD: Non-communicable disease
UPF: Ultra-processed food

## Data availability

No datasets were generated or analysed during the current study.
