# Correction: An unbiased, sustainable, evidence-informed Universal Food Guide: a timely template for national food guides

**DOI:** 10.1186/s12937-024-01043-y

**Published:** 2024-11-15

**Authors:** Elizabeth Dean, Jia Xu, Alice Yee‑Men Jones, Mantana Vongsirinavarat, Constantina Lomi, Pintu Kumar, Etienne Ngeh, Maximilian A. Storz

**Affiliations:** 1 Faculty of Medicine, Department of Physical Therapy, University of British Columbia, Vancouver, BC Canada; 2 Healing Without Medicine, Shenzhen, China; 3 Physicians Committee for Responsible Medicine, Washington, USA; 4 School of Health & Rehabilitation Sciences, The University of Queensland, Brisbane, Australia; 5 Mahidol University, Bangkok, Thailand; 6 Karolinska University Hospital, Stockholm, Sweden; 7 All India Institute of Medical Sciences, New Delhi, India; 8 Louis University Institute, Douala, Cameroon; 9 Research Organisation for Health Education and Rehabilitation, Guideline International Network African Regional Community, Yaoundé, Cameroon; 10 Department of Internal Medicine II, Centre for Complementary Medicine, Medical Center, Faculty of Medicine, University of Freiburg, Freiburg, Germany

**Correction: Nutr J 23, 126 (2024).**

10.1186/s12937-024-01018-z

Following publication of the original article [1], the authors reported that the Reference to a Table 5 within Table 3 of the Supplemental Material, should be to Table 2 that appears within the text of the article.

Included here is the correct Supplemental Material and the original article has been updated.

## Electronic supplementary material

Below is the link to the electronic supplementary material.

Supplementary Material 1
