# Supplementary material for: An unbiased, sustainable, evidence-informed Universal Food Guide: a timely template for national food guides
Source: Nutr J. 2024 Oct 18;23:126. doi: 10.1186/s12937-024-01018-z (PMC11487974; doi:10.1186/s12937-024-01018-z)
Supplement: Supplementary file 1 — Supplementary Material 1. [file 12937_2024_1018_MOESM1_ESM.pdf]

Table 1: Animal-sourced foods and evidence supporting associated health risks

Animal-sourced Foods	Evidence Supporting Health Risks Associated with Consumption of Each Category of Animal-sourced Food
Meat including processed meat and protein	International Agency for Research on Cancer. World Health Organization. IARC Monograph on the Identification of Carcinogenic Hazards to Humans. Agents Classified by the IARC Monographs, Volumes 1-132. Available at: https://monographs.iarc.who.int/agents-classified-by-the-iarc/. (Processed meats are Class 1 carcinogens; Red meat is a Class 2 carcinogen)<br>Naghshi S, Sadeghi O, Willett WC, Esmaillzadeh A. Dietary intake of total, animal, and plant proteins and risk of all cause, cardiovascular, and cancer mortality: systematic review and dose-response meta-analysis of prospective cohort studies. BMJ. 2020 Jul 22;370:m2412. doi: 10.1136/bmj.m2412.<br>Battaglia Richi E, Baumer B, Conrad B, Darioli R, Schmid A, Keller U. Health risks associated with meat consumption: A review of epidemiological studies. Int J Vitam Nutr Res. 2015;85(1-2):70-8.<br>Papier K, Fensom GK, Knuppel A, Appleby PN, Tong TYN, Schmidt JA, et al. Meat consumption and risk of 25 common conditions: outcome-wide analyses in 475,000 men and women in the UK Biobank study. BMC Med. 2021 Mar 2;19(1):53. doi: 10.1186/s12916-021-01922-9.<br>Li Y, Pei H, Zhou C, Lou Y. Dietary cholesterol consumption and incidence of type 2 diabetes mellitus: A dose-response meta-analysis of prospective cohort studies. Nutr Metab Cardiovasc Dis. 2023;33(1):2-10.<br>van Nielen M, Feskens EJ, Mensink M, Sluijs I, Molina E, Amiano P, Ardanaz E, et al. Dietary protein intake and incidence of type 2 diabetes in Europe: the EPIC-InterAct Case-Cohort Study. Diabetes Care. 2014;37(7):1854-62.<br>Ritchie H. Less meat is nearly always better than sustainable meat, to reduce your carbon footprint. 2020. Available at: https://ourworldindata.org/less-meat-or-sustainable-meat<br>Ferdowsian HR, Barnard ND Effects of plant-based diets on plasma lipids. Am J Cardiol. 2009;104(7):947-56.<br>Ashton E, Ball M. Effects of soy as tofu vs meat on lipoprotein concentrations. Eur J Clin Nutr. 2000;54(1):14-9.<br>Ashton EL, Dalais FS, Ball MJ. Effect of meat replacement by tofu on CHD risk factors including copper induced LDL oxidation. J Am Coll Nutr. 2000;19(6):761-7.<br>Carroll KK, Giovannetti PM, Huff MW, Moase O, Roberts DC, Wolfe BM. Hypocholesterolemic effect of substituting soybean protein for animal protein in the diet of healthy young women. Am J Clin Nutr. 1978;31(8):1312-21.<br>Shorey RL, Baza Bn, Lo GS, Steinke FH. Determinants of hypocholesterolemic response to soy and animal protein-based diets. Am J Clin Nutr. 1981;34(9):1769-78.<br>Wiebe SL, Bruce VM, McDonald BE. A comparison of the effect of diets containing beef protein and plant proteins on blood lipids of healthy young men. Am J Clin Nutr. 1984;40(5):982-9.<br>Clifton PM. Protein and coronary heart disease: the role of different protein sources. Curr Atheroscler Rep. 2011;13(6):493-8.

	Fan M, Li Y, Wang C, Mao Z, Zhou W, Zhang L. Dietary protein consumption and the risk of type 2 diabetes: A dose-response meta-analysis of prospective studies. Nutrients. 2019;11(11):2783. doi: 10.3390/nu11112783.<br>Chen Z, Glisic M, Song M, Aliahmad HA, Zhang X, Moumdjian AC, et al. Dietary protein intake and all-cause and cause-specific mortality: results from the Rotterdam Study and a meta-analysis of prospective cohort studies. Eur J Epidemiol. 2020;35(5):411-29.<br>Malik VS, Li Y, Tobias DK, Pan A, Hu FB. Dietary protein intake and risk of type 2 diabetes in US men and women. Am J Epidemiol. 2016;183(8):715-28.<br>Yiqing Song, JoAnn E. Manson, Julie E. Buring, Simin Liu; A prospective study of red meat consumption and type 2 diabetes in middle-aged and elderly women: The Women's Health Study. Diabetes Care. 2004;27(9):2108–15.<br>Pan A, Sun Q, Bernstein AM, Manson JE, Willett WC, Hu FB. Changes in red meat consumption and subsequent risk of type 2 diabetes mellitus: three cohorts of US men and women. JAMA Intern Med. 2013;173(14):1328-35.<br>You, W., Henneberg, M. Meat consumption providing a surplus energy in modern diet contributes to obesity prevalence: an ecological analysis. BMC Nutr. 2016;2,22. doi.org/10.1186/s40795-016-0063-9.<br>Gatarek P, Kaluzna-Czaplinska J. Trimethylamine N-oxide (TMAO) in human health. EXCLI J. 2021;20:301-19.<br>Barnard N, Levin S, Trapp C. Meat consumption as a risk factor for type 2 diabetes. Nutrients. 2014;6(2):897-910.<br>Pan A, Sun Q, Bernstein AM, Schulze MB, Manson JE, Willett WC, et al. Red meat consumption and risk of type 2 diabetes: 3 cohorts of US adults and an updated meta-analysis. Am J Clin Nutr. 2011 Oct;94(4):1088-96.<br>Clarke R, Frost C, Collins R, Appleby P, Peto R. Dietary lipids and blood cholesterol: quantitative meta-analysis of metabolic ward studies. BMJ. 1997;314(7074):112-17.<br>Howell WH, McNamara DJ, Tosca MA, Smith BT, Gaines JA. Plasma lipid and lipoprotein responses to dietary fat and cholesterol: a meta-analysis. Am J Clin Nutr. 1997;65(6):1747-64.<br>Hopkins PN. Effects of dietary cholesterol on serum cholesterol: a meta-analysis and review. Am J Clin Nutr. 1992;55(6):1060-70.<br>Levin S, Wells C, Barnard N. Dietary cholesterol and blood cholesterol concentrations. JAMA. 2015;314(19):2083-4.<br>Berger S, Raman G, Vishwanathan R, Jacques PF, Johnson EJ. Dietary cholesterol and cardiovascular disease: a systematic review and meta-analysis. Am J Clin Nutr. 2015;102(2):276-94.<br>Tappel A. Heme of consumed red meat can act as a catalyst of oxidative damage and could initiate colon, breast and prostate cancers, heart disease and other diseases. Med Hypotheses. 2007;68(3):562-4.<br>Gatarek P, Kaluzna-Czaplinska J. Trimethylamine N-oxide (TMAO) in human health. EXCLI J. 2021;20:301-19.<br>Wang M, Tang WHW, Li XS, de Oliveira Otto MC, Lee Y, Lemaitre RN, et al. The gut microbial metabolite trimethylamine N-oxide, incident CKD, and kidney function decline. J Am Soc Nephrol. 2024 Apr 9. doi: 10.1681/ASN.0000000000000344.<br>Wang Z, Bergeron N, Levison BS, Li XS, Chiu S, Jia X, et al. Impact of chronic dietary red meat, white meat, or non-meat protein on trimethylamine N-oxide metabolism and renal excretion in healthy men and women, Eur Heart J. 2019;40,(7):583–94.

	Wang Z, Klipfell E, Bennett BJ, Koeth R, Levison BS, Dugar B, et al. Gut flora metabolism of phosphatidylcholine promotes cardiovascular disease. Nature. 2011;472(7341):57–63.<br>InterAct Consortium; Bendinelli B, Palli D, Masala G, Sharp SJ, Schulze MB, Guevara M, et al. Association between dietary meat consumption and incident type 2 diabetes: the EPIC-InterAct study. Diabetologia. 2013;56(1):47-59.<br>Lippi G, Mattiuzzi C, Cervellin G. Meat consumption and cancer risk: a critical review of published meta-analyses. Crit Rev Oncol Hematol. 2016;97:1-14.<br>Huang Y, Cao D, Chen Z, Chen B, Li J, Guo J, et al. Red and processed meat consumption and cancer outcomes: Umbrella review. Food Chem. 2021 Sep 15;356:129697. doi: 10.1016/j.foodchem.2021.129697.<br>Farvid MS, Sidahmed E, Spence ND, Mante Angua K, Rosner BA, Barnett JB. Consumption of red meat and processed meat and cancer incidence: a systematic review and meta-analysis of prospective studies. Eur J Epidemiol. 2021;36(9):937-51.<br>Jensen TK, Heitmann BL, Jensen MB, Halldorsson TI, Andersson AM, Skakkebaek, NE, et al. High dietary intake of saturated fat is associated with reduced semen quality among 701 young Danish men from the general population. Am J Clin Nutr. 2013;97(2):411-8.<br>Padron RS, Mas J, Zamora R, Riverol F, LIcea M, Mallea L, et al. Lipids and testicular function. Int Urol Nephrol. 1989;21(5):515-9.<br>Afeiche MC, Williams PL, Gaskins AJ, Mendiola J, Jorgensen N, Swan SH, et al. Meat intake and reproductive parameters among men. Epidemiology. 2014;25(3):323-30.<br>Schisterman EF, Mumford SL, Chen Z, Browne RW, Boyd Barr D, Kim S, et al. Lipid concentrations and semen quality: the LIFE study. Andrology. 2014;2(3):408-15.
Poultry	Maki KC, Van Elswyk ME, Alexander DD, Rains TM, Sohn EL, McNeill S. A meta-analysis of randomized controlled trials that compare the lipid effects of beef versus poultry and/or fish consumption. J Clin Lipidol. 2012;6(4):352-61.<br>Y Wang, C Lehane, K Ghebremeskel, M A Crawford. Modern organic and broiler chickens sold for human consumption provide more energy from fat than protein. Public Health Nutr. 2010;13(3):400-8.<br>Gilsing AMJ, Weijenberg MP, Hughes LAE, Ambergen T, Dagnelie PC, Goldbohm RA, et al. Longitudinal changes in BMI in older adults are associated with meat consumption differentially, by type of meat consumed. Nutr. 2012;142(2):340-9.<br>Bergeron N, Chiu S, Williams PT, King SM, Krauss RM. Effects of red meat, white meat, and nonmeat protein sources on atherogenic lipoprotein measures in the context of low compared with high saturated fat intake: a randomized controlled trial. Am J Clin Nutr. 2019;110(1):24-33.<br>Chai SJ, Cole D, Nisler A, Mahon BE. Poultry: the most common food in outbreaks with known pathogens, United States, 1998-2012. Epidemiol Infect. 2017;145(2):316-25.<br>Liu CM, Stegger M, Aziz M, Johnson TJ, Waits K, Nordstrom L, Gauld L, et al. Escherichia coli ST131-*H*22 as a foodborne uropathogen. mBio. 2018 Aug 28;9(4):e00470-18. doi: 10.1128/mBio.00470-18.

Dairy	Storhaug CL, Fosse SK, Fadnes LT. Country, regional, and global estimates for lactose malabsorption in adults: a systematic review and meta-analysis. Lancet Gastroenterol Hepatol. 2017;2(10):738-46.<br>Willett WC, Ludwig DS. Milk and health. N Engl J Med. 2020 Feb 13;382(7):644-54.<br>Physicians Committee for Responsible Medicine. Health Concerns About Dairy. Fact Sheet. (Available online at: www.pcrm.org/good-nutrition/nutrition-information/health-concerns-about-dairy; accessed January 14, 2024).<br>Sveiven SN, Bookman R, Ma J, Lyden E, Hanson C, Nordgren TM. Milk consumption and respiratory function in asthma patients: NHANES Analysis 2007-2012. Nutrients. 2021 Apr 2;13(4):1182. doi: 10.3390/nu13041182.<br>Michaëlsson K, Wolk A, Langenskiöld S, Basu S, Warensjö Lemming E, Melhus H, et al. Milk intake and risk of mortality and fractures in women and men: cohort studies. BMJ. 2014 Oct 28;349:g6015. doi: 10.1136/bmj.g6015.<br>Mishra S, Baruah K, Malik VS, Ding EL. Dairy intake and risk of hip fracture in prospective cohort studies: non-linear algorithmic dose-response analysis in 486 950 adults. J Nutr Sci. 2023;12:e96. doi: 10.1017/jns.2023.63.<br>Winters E. The shocking reason why humans started drinking cows' milk. Historic film footage. www.youtube.com/watch?v=qXiu2kjQOJw<br>Jiang W, Ju C, Jiang H, Zhang D. Dairy foods intake and risk of Parkinson's disease: a dose-response meta-analysis of prospective cohort studies. Eur J Epidemiol. 2014;29(9):613-9.<br>López-Plaza B, Bermejo LM, Santurino C, Cavero-Redondo I, Álvarez-Bueno C, Gómez-Candela C. Milk and dairy product consumption and prostate cancer risk and mortality: An overview of systematic reviews and meta-analyses. Adv Nutr. 2019;10(suppl_2):S212-23.<br>Kakkoura MG, Du H, Guo Y, Yu C, Yang L, Pei P, et al.; China Kadoorie Biobank (CKB) Collaborative Group. Dairy consumption and risks of total and site-specific cancers in Chinese adults: an 11-year prospective study of 0.5 million people. BMC Med. 2022 May 6;20(1):134. doi: 10.1186/s12916-022-02330-3.<br>Arora RB, Saxena KN, Choudhury MR, Choudhury RR. Sperm studies in Indian men. Fertil Steril. 1961;12:365-7.<br>Maruyama K, Oshima T, Ohyama K. Exposure to exogenous estrogen through intake of commercial milk produced from pregnant cows. Pediatr Int. 2010;52(1):33-8.<br>Afeiche MC, Bridges ND, Williams PL, Gaskins AJ, Tanrikut C, Petrozza JC, et al. Dairy intake and semen quality among men attending a fertility clinic. Fertil Steril. 2014;101(5):1280-7.<br>Afeiche M, Williams PL, Mendiola J, Gaskins AJ, Jorgensen N, Swan SH, et al. Dairy food intake in relation to semen quality and reproductive hormone levels among physically active young men. Hum Reprod. 2013;28(8):2265-75.<br>Ganmaa D, Wang PY, Qin LQ, Hoshi K, Sato A. Is milk responsible for male reproductive disorders? Med Hypotheses. 2001;57(4):510-4.<br>Daxenberger A, Ibarreta D, Meyer HH. Possible health impact of animal oestrogens in food. Hum Reprod Update. 2001;7(3):340-55.<br>Malekinejad H, Rezabakhsh A. Hormones in dairy foods and their impact on public health - A narrative review article. Iran J Public Health. 2015;44(6):742-58.

	Rozati R, Reddy PP, Reddanna P, Mujtaba R. Role of environmental estrogens in the deterioration of male factor fertility. Fertil Steril. 2002;78(6):1187-94.<br>Yager JD, Davidson NE. Estrogen carcinogenesis in breast cancer. N Engl J Med. 2006;354(3):270-82.<br>Guasch-Ferré M, Becerra-Tomás N, Ruiz-Canela M, Corella D, Schröder H, Estruch R, et al. Total and subtypes of dietary fat intake and risk of type 2 diabetes mellitus in the Prevención con Dieta Mediterránea (PREDIMED) study. Am J Clin Nutr. 2017;105(3):723-35.<br>Fiol M, Serra-Majem L, Lapetra J, Basora J, Martín-Calvo N, Portoles O, et al. Total and subtypes of dietary fat intake and risk of type 2 diabetes mellitus in the Prevención con Dieta Mediterránea (PREDIMED) study. Am J Clin Nutr. 2017;105(3):723-35.
Eggs	Wang Y, Li M, Shi Z. Higher egg consumption associated with increased risk of diabetes in Chinese adults - China Health and Nutrition Survey. Br J Nutr. 2021 Jul 14;126(1):110-17.<br>Zhao B, Gan L, Graubard BI, Männistö S, Albanes D, Huang J. Associations of dietary cholesterol, serum cholesterol, and egg consumption with overall and cause-specific mortality: Systematic review and updated meta-analysis. Circulation. 2022 May 17;145(20):1506-20.<br>Physicians Committee for Responsible Medicine. Health Concerns with Eggs. Fact Sheet. Available at: https://www.pcrm.org/good-nutrition/nutrition-information/health-concerns-with-eggs<br>Li Y, Zhou C, Zhou X, Li L. Egg consumption and risk of cardiovascular diseases and diabetes: a meta-analysis. Atherosclerosis. 2013;229(2):524-30.<br>Spence JD, Jenkins DJ, Davignon J. Dietary cholesterol and egg yolks: not for patients at risk of vascular disease. Can J Cardiol. 2010;26(9):e336-e339. doi: 10.1016/s0828-282x(10)70456-6.<br>U.S. Department of Health and Human Services. Your Guide to Lowering Your Cholesterol with TLC. National Institutes of Health. National Heart, Lung and Blood Institute. Available at: https://www.nhlbi.nih.gov/files/docs/public/heart/chol_tlc.pdf<br>Clarke R, Frost C, Collins R, Appleby P, Peto R. Dietary lipids and blood cholesterol: quantitative meta-analysis of metabolic ward studies. BMJ. 1997;314(7074):112-17.<br>Li Y, Y,Zhou C,Zhou X,Li L. Egg consumption and risk of cardiovascular diseases and diabetes: a meta-analysis. Atherosclerosis. 2013;229(2):524-30.<br>Spence JD, Jenkins DJ, Davignon J. Dietary cholesterol and egg yolks: not for patients at risk of vascular disease. Can J Cardiol. 2010;26(9):e336-e39.<br>Choi Y, Chang Y, Lee JE, Chun S, Cho J, Sung E, et al. Egg consumption and coronary artery calcification in asymptomatic men and women. Atherosclerosis. 2015 Aug;241(2):305-12.<br>Djoussé L, Khawaja OA, Gaziano JM. Egg consumption and risk of type 2 diabetes: a meta-analysis of prospective studies. Am J Clin Nutr. 2016;103(2):474-80.

	Djoussé L, Gaziano JM. Egg consumption in relation to cardiovascular disease and mortality: the Physicians' Health Study. Am J Clin Nutr. 2008;87(4):964-69.<br>Keum N, Lee DH, Marchand N, et al. Egg intake and cancers of the breast, ovary and prostate: a dose-response meta-analysis of prospective observational studies. Br J Nutr. 2015;114(7):1099-107.<br>Tse G, Eslick GD. Egg consumption and risk of GI neoplasms: dose-response meta-analysis and systematic review. Eur J Nutr.2014;53(7):1581-90.<br>Miller CA, Corbin KD, da Costa KA, et al. Effect of egg ingestion on trimethylamine-N-oxide production in humans: a randomized, controlled, dose-response study. Am J Clin Nutr. 2014;100(3):778-86.<br>Zhang J,Zhao Z,Berkel HJ. Egg consumption and mortality from colon and rectal cancers: an ecological study. Nutr Cancer. 2003;46(2):158-65.<br>Stephany RW. Hormonal growth promoting agents in food producing animals. Handb Exp Pharmacol. 2010;(195):355-67.

Fish and Seafood	Gatarek P, Kaluzna-Czaplinska J. Trimethylamine N-oxide (TMAO) in human health. EXCLI J. 2021;20:301-19.<br>Wang Z, Tang WHW, O'Connell T, Garcia E, Jeyarajah EJ, Li XS, Jia X, Weeks TL, Hazen SL. Circulating trimethylamine N-oxide levels following fish or seafood consumption. Eur J Nutr. 2022 Aug;61(5):2357-64.<br>Ku HH, Lin P, Ling MP. Assessment of potential human health risks in aquatic products based on the heavy metal hazard decision tree. BMC Bioinformatics. 2022 Feb 17;22(Suppl 5):620. doi: 10.1186/s12859-022-04603-3.<br>Barbo N, Stoiber T, Naidenko OV, Andrews DQ. Locally caught freshwater fish across the United States are likely a significant source of exposure to PFOS and other perfluorinated compounds. Environ Res. 2023 Mar 1;220:115165. doi: 10.1016/j.envres.2022.115165.<br>International Agency for Research on Cancer. World Health Organization. IARC Monograph on the Identification of Carcinogenic Hazards to Humans. Agents Classified by the IARC Monographs, Volumes 1-132. Available at: https://monographs.iarc.who.int/agents-classified-by-the-iarc/. (Salted fish is a Class 1 carcinogen)<br>Soleha U, Qomaruddin MB. Saltwater fish consumption pattern and incidence of hypertension in adults: A study on the population of Gresik coast, Indonesia. J Public Health Res. 2020 Jul 3;9(2):1846. doi: 10.4081/jphr.2020.1846.<br>Zeng G, You D, Ye L, Wu Y, Shi H, Lin J, Jiang Z, Wei J. n-3 PUFA poor seafood consumption is associated with higher risk of gout, whereas n-3 PUFA rich seafood is not: NHANES 2007-2016. Front Nutr. 2023 Apr 4;10:1075877. doi: 10.3389/fnut.2023.1075877.<br>Health concerns about fish. Physicians Committee for Responsible Medicine. Available at: https://p.widencdn.net/zsvtil/Health-Concerns-About-Fish-Fact-Sheet

	Fatkin D, Cox CD, Martinac B. Fishing for links between omega-3 fatty acids and atrial fibrillation. Circulation. 2022;145(14):1037-1039.

Table 2: Constituents (other than synthetic and other additives) commonly found in processed and ultra-processed foods (NOVA Classifications 2 and 3) and documented health risks

Constituent	Evidence Supporting Health Risks Related to Consumption of Each Constituent
Oils	Zong G, Li Y, Sampson L, Dougherty LW, Willett WC, Wanders AJ, et al. Monounsaturated fats from plant and animal sources in relation to risk of coronary heart disease among US men and women. Am J Clin Nutr. (2018) 107(3): 445–53.<br>Summerhill V, Karagodin V, Grechko A, Myasoedova V, Orekhov A. Vasculoprotective role of olive oil compounds via modulation of oxidative stress in atherosclerosis. Front Cardiovasc Med. 2018 Dec 21;5:188. doi: 10.3389/fcvm.2018.00188.<br>Vogel RA, Corretti MC, Plotnick GD. Effect of a single high-fat meal on endothelial function in healthy subjects. Am J Cardiol. (1997) 79(3):350-4.
Fats	Barnard ND, Alwarith J, Rembert E, Brandon L, Nguyen M, Goergen A, et al. A Mediterranean diet and low-fat vegan diet to improve body weight and cardiometabolic risk factors: A randomized, cross-over trial. J Am Nutr Assoc. 2022;41(2):127-39.<br>Barnard ND, Cohen J, Jenkins DJ, Turner-McGrievy G, Gloede L, Jaster B, et al. A low-fat vegan diet improves glycemic control and cardiovascular risk factors in a randomized clinical trial in individuals with type 2 diabetes. Diabetes Care. 2006 Aug;29(8):1777-83.<br>Hodson L, Skeaff CM, Chisholm WA. The effect of replacing dietary saturated fat with polyunsaturated or monounsaturated fat on plasma lipids in free-living young adults. Eur J Clin Nutr. 2001;55(10):908-15.<br>Vogel RA, Corretti MC, Plotnick GD. Effect of a single high-fat meal on endothelial function in healthy subjects. Am J Cardiol. (1997) 79(3):350-4.
Sodium and Salt	Note: The total sodium shown on the Nutrition Facts label includes the sodium from salt, plus the sodium from any other sodium-containing ingredient in the product. For example, this includes preservative ingredients such as sodium nitrate, sodium citrate, monosodium glutamate (MSG) or sodium benzoate.<br>Sodium Intake and Health (cdc.gov). Available at: www.cdc.gov/salt/index.htm<br>Bibbins-Domingo K, Chertow GM, Coxson PG, Moran A, Lightwood JM, Pletcher MJ, et al. Projected effect of dietary salt reductions on future cardiovascular disease. N Engl J Med. 2010;362(7):590-9.<br>Cogswell ME, Zhang Z, Carriquiry AL, Gunn JP, Kuklina EV, Saydah SH, et al. Sodium and potassium intakes among US adults: NHANES 2003-2008. Am J Clin Nutr. 2012;96(3):647-57.<br>Malta D, Petersen KS, Johnson C, Trieu K, Rae S, Jefferson K, et al. High sodium intake increases blood pressure and risk of kidney disease. From the Science of Salt: A regularly updated systematic review of salt and health outcomes (August 2016 to March 2017). J Clin Hypertens (Greenwich). 2018;20(12):1654–65.<br>Allison A, Fouladkhah A. Adoptable interventions, human health, and food safety Considerations for reducing sodium content of processed food products. Foods. 2018;7(2):16. doi: 10.3390/foods7020016.

	Carrillo-Larco RM, Bernabe-Ortiz A. Sodium and salt consumption in Latin America and the Caribbean: A systematic-review and meta-analysis of population-based studies and surveys. Nutrients. 2020;2(2):556. doi: 10.3390/nu12020556.<br>Fu Q, Chen R, Ding Y, Xu S, Huang C, He B, et al. Sodium intake and the risk of various types of cardiovascular diseases: a Mendelian randomization study. Front Nutr. 2023;10:1250509. doi: 10.3389/fnut.2023.1250509.<br>McLaren L, Sumar N, Barberio AM, Trieu K, Lorenzetti DL, Tarasuk V, Webster J, et al. Population-level interventions in government jurisdictions for dietary sodium reduction. Cochrane Database Syst Rev. 2016 Sep 16;9(9):CD010166. doi: 10.1002/14651858.CD010166.pub2.<br>Wang YJ, Yeh TL, Shih MC, Tu YK, Chien KL. Dietary sodium intake and risk of cardiovascular disease: A systematic review and dose-response meta-analysis. Nutrients. 2020 Sep 25;12(10):2934. doi: 10.3390/nu12102934.<br>Greenwood H, Barnes K, Clark J, Ball L, Albarqouni L. Long-term effect of salt substitution for cardiovascular outcomes : A systematic review and meta-analysis. Ann Intern Med. 2024 Apr 9. doi: 10.7326/M23-2626.
Sugar (Often disguised using over 60 different names)	EFSA Panel on Nutrition, Novel Foods and Food Allergens (NDA); Turck D, Bohn T, Castenmiller J, de Henauw S, Hirsch-Ernst KI, Knutsen HK, et al. Tolerable upper intake level for dietary sugars. EFSA J. 2022 Feb 28;20(2):e07074. doi: 10.2903/j.efsa.2022.7074.<br>Huang Y, Chen Z, Chen B, Li J, Yuan X, Li J, et al. Dietary sugar consumption and health: umbrella review. BMJ. 2023 Apr 5;381:e071609. doi: 10.1136/bmj-2022-071609.<br>Ma X, Nan F, Liang H, Shu P, Fan X, Song X, et al. Excessive intake of sugar: An accomplice of inflammation. Front Immunol. 2022 Aug 31;13:988481. doi: 10.3389/fimmu.2022.988481.<br>Huang Y, Chen Z, Chen B, Li J, Yuan X, Li J, et al. Dietary sugar consumption and health: umbrella review. BMJ. 2023 Apr 5;381:e071609. doi: 10.1136/bmj-2022-071609.<br>Ricciuto L, Fulgoni III VL, Gaine PC, Scott M0, DiFrancesco L. Trends in added sugars intake and sources among US children, adolescents, and teens using NHANES 2001-2018. J Nutr. 2022;152(2):568–78.<br>Huneault HE, Ramirez Tovar A, Sanchez-Torres C, Welsh JA, Vos MB. The impact and burden of dietary sugars on the liver. Hepatol Commun. 2023 Nov 6;7(11):e0297. doi: 10.1097/HC9.0000000000000297.<br>Malik VS, Hu FB. The role of sugar-sweetened beverages in the global epidemics of obesity and chronic diseases. Nat Rev Endocrinol. 2022;18(4):205-18.<br>World Health Organization. Guidelines: sugars intake for adults and children. Available at: http://www.who.int/nutrition/publications/guidelines/sugars_intake/en/<br>Scientific Advisory Committee on Nutrition. Carbohydrates and Health Report. London, England: The Stationery Office; 2015. Available at: https://assets.publishing.service.gov.uk/media/5a7f7cc3ed915d74e622ac2a/SACN_Carbohydrates_and_Health.pdf

	Imamura F, O'Connor L, Ye Z, Mursu J, Hayashino Y, Bhupathiraju SN, Forouhi NG. Consumption of sugar sweetened beverages, artificially sweetened beverages, and fruit juice and incidence of type 2 diabetes: systematic review, meta-analysis, and estimation of population attributable fraction. BMJ. 2015 Jul 21;351:h3576. doi: 10.1136/bmj.h3576.<br>Gulati S, Misra A. Sugar intake, obesity, and diabetes in India. Nutrients. 2014;6(12):5955-74.<br>University of California at San Francisco. Hidden in Plain Sight. There are at least 61 different names for sugar listed on food labels and sugar is hiding in 74% of packaged foods. Available at: www.sugarscience.ucsf.edu/hidden-in-plain-sight/<br>Yudkin J. Pure, White and Deadly. How Sugar is Killing Us and What We Can Do to Stop It. Penguin Books, Ltd.:London, UK. 1972.

Table 3: Evidence supporting health-harming risks of The Dietary Guidelines for Americans (DGA)
Source: https://www.dietaryguidelines.gov/sites/default/files/2021-03/Dietary_Guidelines_for_Americans-2020-2025.pdfx

The DGA describes a healthy dietary pattern as one that:

**Constituents**<br>**Evidence Supporting Health-harming Effects**<br>**(*Italicized* constituents are those with evidence of health-harming effects)**	**Evidence Supporting Health-harming Effects of Each Constituent**
Includes a variety of vegetables; fruits; grains (at least half whole grains);	Substantial health-benefitting evidence (see Table 2 in the article)
fat-free and low-fat milk, yogurt, and cheese; and oils.	Also see Table 1, Dairy<br>Willett WC, Ludwig DS. Milk and Health. N Engl J Med. 2020;382(7):644-54.<br>Zong G, Li Y, Sampson L, Dougherty LW, Willett WC, Wanders AJ, et al. Monounsaturated fats from plant and animal sources in relation to risk of coronary heart disease among US men and women. Am J Clin Nutr. 2018;107(3):445–53.<br>Ferraro PM, Bargagli M, Trinchieri A, Gambaro G. Risk of kidney stones: Influence of dietary factors, dietary patterns, and vegetarian-vegan diets. Nutrients. 2020 Mar 15;12(3):779. doi: 10.3390/nu12030779.<br>Li M, Shen M, Lu J, Yang J, Huang Y, Liu L, Fan H, Xie J, Xie M. Maillard reaction harmful products in dairy products: Formation, occurrence, analysis, and mitigation strategies. Food Res Int. 2022 Jan;151:110839. doi: 10.1016/j.foodres.2021.110839.
Milk and milk products are good sources of vitamin B12. Many ready-to-eat breakfast cereals are fortified with vitamin B12.	Note: Although vitamin B12 may be found in animal-sourced foods, this does not suggest that these sources are ideal for humans.<br>Ready-to-eat breakfast cereals may be fortified with vitamin B12, but these may also be high in fat, sugar and salt, and a range of preservatives.<br>Wang XJ, Jiang CQ, Zhang WS, Zhu F, Jin YL, Woo J, et al. Milk consumption and risk of mortality from all-cause, cardiovascular disease and cancer in older people. Clin Nutr. 2020;39(11):3442-51.

	Kaufman EJ, Tan C. White as milk: Biocentric bias in the framing of lactose intolerance and lactase persistence. Sociol Health Illn. 2022;44(9):1533-50.<br>Physicians Committee for Responsible Medicine. Health Concerns About Dairy. Fact Sheet. Available at: www.pcrm.org/good-nutrition/nutrition-information/health-concerns-about-dairy
Includes a variety of protein foods such as lean meats; poultry; eggs; seafood;	Also see Table 1, Meat; Poultry; Eggs; and Fish and Seafood<br>International Agency for Research on Cancer. World Health Organization. IARC Monograph on the Identification of Carcinogenic Hazards to Humans. Agents Classified by the IARC Monographs, Volumes 1-132. Available at: https://monographs.iarc.who.int/agents-classified-by-the-iarc/<br>Di Y, Ding L, Gao L, Huang H. Association of meat consumption with the risk of gastrointestinal cancers: a systematic review and meta-analysis. BMC Cancer. 2023 Aug 23;23(1):782. doi: 10.1186/s12885-023-11218-1.<br>Papier K, Fensom GK, Knuppel A, Appleby PN, Tong TYN, Schmidt JA, et al. Meat consumption and risk of 25 common conditions: outcome-wide analyses in 475,000 men and women in the UK Biobank study. BMC Med. 2021 Mar 2;19(1):53. doi: 10.1186/s12916-021-01922-9.<br>Chao A, Thun MJ, Connell CJ, McCullough ML, Jacobs EJ, Flanders WD, et al. Meat consumption and risk of colorectal cancer. JAMA. 2005;293(2):172-82.<br>Zhang M, Hou ZK, Huang ZB, Chen XL, Liu FB. Dietary and lifestyle factors related to gastroesophageal reflux disease: A systematic review. Ther Clin Risk Manag. 2021;17:305-23.<br>Battaglia Richi E, Baumer B, Conrad B, Darioli R, Schmid A, Keller U. Health risks associated with meat consumption: A review of epidemiological studies. Int J Vitam Nutr Res. 2015;85(1-2):70-8.<br>Li Y, Pei H, Zhou C, Lou Y. Dietary cholesterol consumption and incidence of type 2 diabetes mellitus: A dose-response meta-analysis of prospective cohort studies. Nutr Metab Cardiovasc Dis. 2023;33(1):2-10.<br>van Nielen M, Feskens EJ, Mensink M, Sluijs I, Molina E, Amiano P, et al.; InterAct Consortium. Dietary protein intake and incidence of type 2 diabetes in Europe: the EPIC-InterAct Case-Cohort Study. Diabetes Care. 2014;37(7):1854-62.

	InterAct Consortium; Bendinelli B, Palli D, Masala G, Sharp SJ, Schulze MB, Guevara M, et al. Association between dietary meat consumption and incident type 2 diabetes: the EPIC-InterAct study. Diabetologia. 2013;56(1):47-59.<br>Ferdowsian HR, Barnard ND. Effects of plant-based diets on plasma lipids. Am J Cardiol. 2009;104(7):947-56.<br>Ashton E, Ball M. Effects of soy as tofu vs meat on lipoprotein concentrations. Eur J Clin Nutr. 2000;54(1):14-9.<br>Ashton EL, Dalais FS, Ball MJ. Effect of meat replacement by tofu on CHD risk factors including copper induced LDL oxidation. J Am Coll Nutr. 2000;19(6):761-7.<br>Carroll KK, Giovannetti PM, Huff MW, Moase O, Roberts DC, Wolfe BM. Hypocholesterolemic effect of substituting soybean protein for animal protein in the diet of healthy young women. Am J Clin Nutr. 1978;31(8):1312-21.<br>Shorey RL, Bazan B, Lo GS, Steinke FH. Determinants of hypocholesterolemic response to soy and animal protein-based diets. Am J Clin Nutr. 1981;34(9):1769-78.<br>Wiebe SL, Bruce VM, McDonald BE. A comparison of the effect of diets containing beef protein and plant proteins on blood lipids of healthy young men. Am J Clin Nutr. 1984;40(5):982-9.<br>Clifton PM. Protein and coronary heart disease: the role of different protein sources. Curr Atheroscler Rep. 2011;13(6):493-8.<br>Barnard N, Levin S, Trapp C. Meat consumption as a risk factor for type 2 diabetes. Nutrients. 2014;6(2):897-910.<br>Zhuang P, Wu F, Mao L, Zhu F, Zhang Y, Chen X, et al. Egg and cholesterol consumption and mortality from cardiovascular and different causes in the United States: A population-based cohort study. PLoS Med. 2021 Feb 9;18(2):e1003508. doi: 10.1371/journal.pmed.1003508.<br>Song M, Fung TT, Hu FB, Willett WC, Longo VD, Chan AT, et al. Association of animal and plant protein intake with all-cause and cause-specific mortality. JAMA Intern Med. 2016;176(10):1453-63.<br>Fontana L, Cummings NE, Arriola Apelo SI, Neuman JC, Kasza I, Schmidt BA, et al. Decreased consumption of branched-chain amino acids improves metabolic health. Cell Rep. 2016;16(2):520-30.<br>Huang J, Liao LM, Weinstein SJ, Sinha R, Graubard BI, Albanes D. Association between plant and animal protein intake and overall and cause-specific mortality. JAMA Intern Med. 2020;180(9):1173-84.

	Qi XX, Shen P. Associations of dietary protein intake with all-cause, cardiovascular disease, and cancer mortality: A systematic review and meta-analysis of cohort studies. Nutr Metab Cardiovasc Dis. 2020;30(7):1094-105.<br>Le LT, Sabaté J. Beyond meatless, the health effects of vegan diets: findings from the Adventist cohorts. Nutrients. 2014;6(6):2131-47.
Beans, peas, and lentils; nuts and seeds; and soy products.	Substantial health-benefitting evidence (see Table 2 in the article)
Fish and red meat are excellent sources of vitamin B12. Poultry and eggs also contain vitamin B12.	Note: Although vitamin B12 may be found in animal-sourced foods, this does not imply that these sources are optimal for humans. Animals are not inherently sources of the vitamin which is obtained through the organisms they ingest. Today, humans are less likely to obtain vitamin B12 from natural sources, thus when on plant-based diets, they require foods fortified with the vitamin or a daily supplement.
Limits foods and beverages higher in added sugars, saturated fat, and sodium.<br><br>*Specifically:*<br>*Limiting added sugars to less than 10% of calories per day for people age 2 and older and avoiding added sugars for infants and toddlers.*<br>*Limiting saturated fat to less than 10% of calories per day at age 2 and older.*<br>*Limiting sodium intake to less than 2,300 mg per day (or less for people younger than 14).*	Evidence supports avoiding these constituents. Sodium in the form of iodized salt is necessary for human health but only <1500 mg daily (<1/3 tsp daily)<br>How Much Sodium Should I Eat a Day? Available at: https://www.heart.org/en/healthy-living/healthy-eating/eat-smart/sodium/how-much-sodium-should-i-eat-per-day<br>Fu Q, Chen R, Ding Y, Xu S, Huang C, He B, et al. Sodium intake and the risk of various types of cardiovascular diseases: a Mendelian randomization study. Front Nutr. 2023 Dec 22;10:1250509. doi: 10.3389/fnut.2023.1250509.<br>Note: Most people are unlikely to know the over 50 names for sugar ingredients; sources of saturated fat vs. other fat; and the distinction between and sources of sodium vs. salt, and that on average 60-80% of sodium that people consume, comes from ultra-processed food, not from the salt shaker.<br>There is a low probability that the average person is able to calculate accurately, the values related to sugars, saturated fat, and sodium in their eating pattern as per the Dietary Guidelines for Americans.<br>Campbell NR, Johnson JA, Campbell TS. Sodium consumption: An individual's choice? Int J Hypertens. 2012;2012:860954. doi: 10.1155/2012/860954.

Limits alcoholic beverages.<br><br>*Specifically: For those that drink alcohol, limiting alcoholic beverages to two drinks or less a day for men and one drink or less a day for women.*	Note: There is no evidence-based limit for safe alcohol consumption.<br>International Agency for Research on Cancer. World Health Organization. IARC Monograph on the Identification of Carcinogenic Hazards to Humans. Agents Classified by the IARC Monographs, Volumes 1-132. Available at: https://monographs.iarc.who.int/agents-classified-by-the-iarc/. (Alcohol is a Class 1 carcinogen)<br>Canada's revised evidence-based, low-risk alcohol guidelines. No adverse effects reported for two drinks or less 'a week' for men or one drink or less 'a week' for women Available at: www.ccsa.ca/canadas-guidance-alcohol-and-health for evidence and drink volumes<br>Arora M, ElSayed A, Beger B, Naidoo P, Shilton T, Jain N, et al. The impact of alcohol consumption on cardiovascular health: myths and measures. Glob Heart. 2022 Jul 22;17(1):45. doi: 10.5334/gh.1132.
Stays within your *daily calorie needs.*	Greger M. How Not to Diet. [Evidence Synthesis Monograph]. Flatiron Books. New York, NY, 2019.<br>Note: Most individuals have no way of assessing their daily calorie needs.<br>Note: Healthy nutrition is quality rather than calories.

Table 4: Evidence supporting the Universal Food Guide consisting of whole-food, low-fat vegan nutrition; such nutrition is rich in fiber, micronutrients, vitamins and minerals, legumes (beans, peas, and lentils that are all rich protein), whole grains, nuts, and vegetables and fruit; and with little to no added sugar, salt and fat, particularly no trans nor saturated fat

Attribute/ Characteristic	Supporting Evidence
Humans, anatomically, physiologically, metabolically resemble herbivores, rather than omnivores, suggesting that they are 'vegan-by-design'; evidence based on comparing over 18 dimensions of anatomy, physiology, metabolism, humans have no similarities to omnivores<br>Humans share 99.9% of DNA pairs, thus are more similar to each other than dissimilar	Mills M. The comparative anatomy of eating. Review. 2019. https:// www.drmiltonmillsplantbasednation.com/the-comparative-anatomy-of-eating/<br>Mills M. Are we designed to eat meat? Review. https://youtu.be/kGDYydkvg3E?si=V2xs9YQUHh6wCd_7<br>Clarys P, Deliens T, Huybrechts I, Deriemaeker P, Vanaelst B, De Keyzer W, et al. Comparison of nutritional quality of the vegan, vegetarian, semi-vegetarian, pesco-vegetarian and omnivorous diet. Nutrients. 2014;6(3):1318-32.<br>Human Genome Variation. National Human Genome Research Institute. Available at: https://www.genome.gov/dna-day/15-ways/human-genomic-variation
• Humans closely resemble their non-human primate relatives: human genome only 1-2% different<br>• Incidence of nutrition-related NCDs* that are common in humans, are rare in our closest	Milton K. Nutritional characteristics of wild primate foods: do the diets of our closest living relatives have lessons for us? Nutrition. 1999;15(6):488-98.<br>Gunter C, Dhand R. The chimpanzee genome. Nature. 2005;437, 47. doi.org/10.1038/436047a.<br>Cayton JB, Vangay P, Huang H, Knights D. Captivity humanizes the primate microbiome. PNAS. 2016;113(37):10376-81.<br>Cabana F, Jasmi R, Maguire R. Great ape nutrition: low-sugar and high-fibre diets can lead to increased natural behaviours, decreased regurgitation and reingestion, and reversal of prediabetes. Int Zoo Yearbook. Available at: https://zslpublications.onlinelibrary.wiley.com/doi/10.1111/izy.12172<br>Sharma AK, Petrzelkova K, Pafco B, Jost Robinson CA, Fuh T, Wilson BA, et al. Traditional human populations and nonhuman primates show parallel gut microbiome adaptations to analogous ecological conditions. mSystems. 2020 Dec 22;5(6):e00815-20. doi: 10.1128/mSystems.00815-20.

relatives living in their natural settings<br>• Conversely, when non-human primates are exposed to elements of the Standard American or Western Diet, nutrition-related NCDs* that are observed in humans, manifest	
• Plant-based, particularly vegan diets are anti-inflammatory, thereby, reduce risk of NCDs* including heart disease, several cancers, hypertension, stroke, type 2 diabetes, obesity, gastrointestinal diseases, autoimmune diseases, renal disease, and Alzheimer's disease; diseases associated with the pro-inflammatory	Report of the EAT Lancet Commission. Healthy Diets from Sustainable Food Systems. Available at: https://eatforum.org/content/uploads/2019/07/EAT-Lancet_Commission_Summary_Report.pdf<br>Buettner D, Skemp S. Blue Zones: Lessons learned from the world's longest lived. Am J Lifestyle Med. 2016:10(5):318-21.<br>Campbell TC, Campbell TM. The China Study [Evidence Based Monograph]. Benbella Books, Inc.:Dallas, TX, 2006.<br>Dawczynski C, Weidauer T, Richert C, Schlattmann P, Dawczynski K, Kiehntopf M. Nutrient intake and nutrition status in vegetarians and vegans in comparison to omnivores - the Nutritional Evaluation (NuEva) Study.<br>Front Nutr. 2022; 9: 819106. doi: 10.3389/fnut.2022.81910.<br>Afshin A, Micha R, Khatibzadeh S, Mozaffarian D. Consumption of nuts and legumes and risk of incident ischemic heart disease, stroke, and diabetes: A systematic review and meta-analysis. Am J Clin Nutr 2014;100:278–88.<br>Nanri A, Mizoue T, Takahashi Y, Kirii, K, Inoue, M, Noda M, et al. Soy product and isoflavone intakes are associated with a lower risk of type 2 diabetes in overweight Japanese women. J Nutr 2010;140:580–86.<br>Ornish D, Brown SE, Scherwitz LW, Billings JH, Armstrong WT, Ports TA, et al. Can lifestyle changes reverse coronary artery disease? The Lifestyle Heart Trial. Lancet. 1990;336:129-33.<br>Ornish D, Ornish A. UnDo It! How Simple Lifestyle Changes Can Reverse Most Chronic Diseases [Evidence Synthesis Monograph]. Ballantyne Books: New York, NY, 2019.<br>Greger M. How Not to Die. [Evidence Synthesis Monograph]. Flatiron Books:New York, NY, 2015.<br>Greger M. How Not to Diet. [Evidence Synthesis Monograph]. Flatiron Books:New York, NY, 2019.<br>Greger M. The Scientific Approach to Getting Healthier as You Get Older. How Not to Age. [Evidence Synthesis Monograph]. Flatiron Books:New York, NY, 2023.

Standard American or Western Diet<br>• Such diets are also associated with lower body mass index and metabolic and inflammatory indices<br>• Ornish and colleagues reported over 30 years ago in Lancet, that ischemic heart disease can be reversed with a low-fat vegan diet, based on objective angiographic evidence	Understanding inflammation. Harvard Medical School Guide. Harvard Health Publishing: Boston, MA, 2018.<br>Ricker MA, Haas WC. Anti-inflammatory diet in clinical practice: A review. Nutr Clin Prac. 2017;32(3):318-25.<br>Zhang S, Stubbendorff A, Olsson K, Ericson U, Niu K, Qi L, et al. Adherence to the EAT-Lancet diet, genetic susceptibility, and risk of type 2 diabetes in Swedish adults. Metabolism. 2023 Apr;141:155401. doi: 10.1016/j.metabol.2023.155401.
The 20-year China Study showed the closer people adhered to plant-based nutrition, the healthier they were compared with those who consumed animal-sourced foods, i.e., beef, pork, poultry, fish, eggs, cheese, and milk; and refined carbohydrates and processed foods<br>Consuming 'any' cholesterol is unhealthy	Campbell TC, Campbell TM. The China Study [Evidence Based Monograph]. Benbella Books, Inc.:Dallas, TX, 2006.<br>Campbell TC, Campbell TM. The China Study. Revised and Expanded. [Evidence Based Monograph]. Benbella Books, Inc.: Dallas.TX, 2016.<br>Esselstyn CB Jr. In cholesterol lowering, moderation kills. Cleve Clin J Med. 2000;67(8):560-4.

The Mediterranean diet, plant-based vs. meat-based has been heralded for years as being the healthiest diet Note: rather than a type of cuisine, the elements of the Mediterranean diet can be incorporated into any cultural cuisine	Curtis BM, O'Keefe JH Jr. Understanding the Mediterranean diet. Could this be the new "gold standard" for heart disease prevention? Postgrad Med. 2002;112(2):35-8 and 41-5.<br>Kastorini CM, Milionis HJ, Esposito K, Giugliano D, Goudevenos JA, Panagiotakos DB. The effect of Mediterranean diet on metabolic syndrome and its components: a meta-analysis of 50 studies and 534,906 individuals. J Am Coll Cardiol. 2011;57(11):1299–313.<br>Soltani S, Jayedi A, Shab-Bidar S, Becerra-Tomás N, Salas-Salvadó J. Adherence to the Mediterranean diet in relation to all-cause mortality: a systematic review and dose-response meta-analysis of prospective cohort studies. Adv Nutr. 2019;10(6):1029–39.<br>The Diet Review. Harvard Medical School Guide. Harvard Health Publishing, Harvard Medical School: Boston, MA, 2020.<br>Ros E, Martínez-González MA, Estruch R, Salas-Salvadó J, Fitó M, Martínez JA, et al. Mediterranean diet and cardiovascular health: Teachings of the PREDIMED study. Adv Nutr. 2014;5(3):330S-36S.
Plant-based nutrition is associated with longer telomeres, a marker of biological aging	D'Angelo S. Diet and Aging: The Role of polyphenol-rich diets in slow down the shortening of telomeres: A review. Antioxidants (Basel). 2023 Dec 7;12(12):2086. doi: 10.3390/antiox12122086.<br>Crous-Bou M, Molinuevo JL, Sala-Vila A. Plant-rich dietary patterns, plant foods and nutrients, and telomere length. Adv Nutr. 2019;10(Suppl_4):S296-S303.<br>Maleki M, Khelghati N, Alemi F, Bazdar M, Asemi Z, Majidinia M, et al. Stabilization of telomere by the antioxidant property of polyphenols: Anti-aging potential. Life Sci. 2020;259:118341. doi: 10.1016/j.lfs.2020.118341.<br>Meccariello R., D'Angelo S. Impact of polyphenolic-food on longevity: An elixir of life. An overview. Antioxidants. 2021;10:507. doi: 10.3390/antiox10040507.
The longest and healthiest living people the world consume predominantly plant-based diets (The Blue Zones)	Buettner D, Skemp S. Blue Zones: Lessons learned from the world's longest lived. Am J Lifestyle Med. 2016:10(5):318-21.<br>Marston HR, Niles-Yokum K, Silva PA. A Commentary on Blue Zones®: A critical review of age-friendly environments in the 21st century and beyond. Int J Environ Res Pub Health. 2021 Jan 19;18(2):837. doi: 10.3390/ijerph18020837.
The whole-food, low-fat vegan diet now surpasses the previous dietary gold standard, the Mediterranean diet with respect to	Barnard ND, Alwarith J, Rembert E, Brandon L, Nguyen M, Goergen A, et al. A Mediterranean diet and low-fat vegan diet to improve body weight and cardiometabolic risk factors: A randomized, cross-over trial. J Am Nutr Assoc. 2022;41(2):127-39.

cardiometabolic outcomes	
The notion of ‘moderation’ (or use of words such as ‘limit’ or ‘reduce’) vis-à-vis unhealthy foods is misleading and implies that a little heart disease is okay, a little high blood pressure is okay, a little Alzheimer’s disease is okay, etc	Esselstyn CB Jr. In cholesterol lowering, moderation kills. Cleve Clin J Med. 2000;67(8):560-4.
Identical twin study showed the twin on the vegan study showed superior cardiometabolic markers just after two months, compared with the twin on the healthy standard Western or American Diet	Landry MJ, Ward CP, Cunanan KM, et al. Cardiometabolic effects of omnivorous vs vegan diets in identical twins: A randomized clinical trial. JAMA Netw Open. 2023 Nov 1;6(11):e2344457. doi: 10.1001/jamanetworkopen.2023.44457.
The number of healthy behaviors practiced is associated with correspondingly less risk of NCDs and optimal self-rated health	Ford ES, Bergmann MM, Kröger J, Schienkiewitz A, Weikert C, Boeing H. Healthy living is the best revenge: findings from the European Prospective Investigation into Cancer and Nutrition-Potsdam study. Arch Intern Med. 2009;169(15):1355-62.<br>Tsai J, Ford ES, Li C, Zhao G, Pearson WS, Balluz LS. Multiple healthy behaviors and optimal self-rated health: findings from the 2007 Behavioral Risk Factor Surveillance System Survey. Prev Med. 2010;51(3-4):268-74.
Leading international dietetic and nutrition	Albert Schweitzer Foundation. Vegan: Healthy Across All Stages of Life Cycle. Available at: https://albertschweitzerfoundation.org/news/vegan-diet-healthy-across-all-stages-of-life-cycle

associations acknowledge that healthy vegan nutrition is healthy across the life cycle (children, pregnant and lactating mothers, and elders); most national dietary guides now acknowledge more plants and less meat	Dietary Guidelines for Americans, 2020-2025;<br>Australian Guide to Healthy Eating for Vegetarians and Vegans (dietitiansaustralia.org.au);<br>British Nutrition Foundation. www.nutrition.org.uk/putting-it-into-practice/plant-based-diets/healthy-eating-for-vegetarians-and-vegans/#:~:text=Healthy%20eating%20for%20vegetarians%20and%20vegans%201%20Well-planned,especially%20wholegrains%20and%20high-fibre%20versions.%20...%20More%20items;<br>Considerations for vegetarian diets - Canada's Food Guide<br>Mangels R. What have the U.S. dietary guidelines said about vegan and vegetarian diets? A look back. Available at: https://www.vrg.org/blog/2023/08/14/what-have-the-u-s-dietary-guidelines-said-about-vegan-and-vegetarian-diets-a-look-back/#:~:text=The%20text%20of%20the%202020,fruits%2C%20vegetables%2C%20and%20nuts
Vegan nutrition is the best for the environment and the planet and is sustainable	Report of the EAT Lancet Commission. Healthy Diets from Sustainable Food Systems. Available at: https://eatforum.org/content/uploads/2019/07/EAT-Lancet_Commission_Summary_Report.pdf<br>Lock K, Smith RD, Dangour AD, Keogh-Brown M, Pigatto G, Hawkes C, et al. Health, agricultural, and economic effects of adoption of healthy diet recommendations. Lancet. 2010;376(9753):1699-709.<br>Craig WJ, Messina V, Rowland I, Frankowska A, Bradbury J, Smetana S, et al. Plant-based dairy alternatives contribute to a healthy and sustainable diet. Nutrients. 2023; 15(15):3393. doi: 10.3390/nu15153393.<br>Johnston JL, Fanzo JC, Cogill B. Understanding sustainable diets: a descriptive analysis of the determinants and processes that influence diets and their impact on health, food security, and environmental sustainability. Adv Nutr. 2014;5(4):418-29.<br>Yanni AE, Iakovidi S, Vasilikopoulou E, Karathanos VT. Legumes: A vehicle for transition to sustainability. Nutrients. 2024;16(1):98. doi: 10.3390/nu16010098.<br>Scarborough P, Clark M, Cobiac L, Papier K, Knuppel A, Lynch J, et al. Vegans, vegetarians, fish-eaters and meat-eaters in the UK show discrepant environmental impacts. Nat Food. 2023;4(7):565-74.
Vegan nutrition is economical for families, for national budgets, and reduced health care costs	Schepers J, Annemans L. The potential health and economic effects of plant-based food patterns in Belgium and the United Kingdom. Nutrition. 2018;48:24-32.<br>Flynn MM, Schiff AR. Economical healthy diets (2012): Including lean animal protein costs more than using extra virgin olive oil. J Hunger Environ Nutr. 2015;10(4):467-82.<br>Office of Health Economics Study: 100% adoption of plant-based diets could save the NHS £6.7 billion a year. Available at: https://www.news-medical.net/documents/final/3-768414-202401080327-Study-100%25-adoption-of-plant-based-diets-could-save-the-NHS-%C2%A367-billion-a-year.pdf

	Connell CL, Zoellner JM, Yadrick MK, Chekuri SC, Crook LB, Bogle ML. Energy density, nutrient adequacy, and cost per serving can provide insight into food choices in the lower Mississippi Delta. J Nutr Educ Behav. 2012;44(2):148-53.<br>Campbell TC. Nutritional renaissance and public health policy. J Nutr Biol. 2017;3(1):124-38.
Vegan nutrition is ethical and a moral choice with respect to planetary health and animal welfare	Pickett S. Veganism, moral motivation and false consciousness. Agric Environ Ethics. 2021;34(3):15. doi: 10.1007/s10806-021-09857-0.<br>Hull SC, Charles J, Caplan AL. Are we what we eat? The moral imperative of the medical profession to promote plant-based nutrition. Am J Cardiol. 2023;188:15-21.<br>Benatar D. The chickens come home to roost. Am J Public Health. 2007 Sep;97(9):1545-6. doi: 10.2105/AJPH.2006.090431.<br>Nuffield Bioethics. Available at: https://www.nuffieldbioethics.org/wp-content/uploads/Animals-Chapter-4-The-Capacity-of-Animals-to-Experience-Pain-Distress-and-Suffering.pdf<br>Winters E. Is there actually an ethical reason not to eat fish? Review. Available at: https://www.youtube.com/watch?v=y8Nj1-YZDlc
Population-based nutrition counselling can be supported based on humans being more similar than dissimilar	American Museum of Natural History. The chimpanzee and bonobo are humans' closest living relatives. Available at: https://theconversation.com/bonobos-and-chimps-what-our-closest-relatives-tell-us-about-humans-202265#:~:text=Among%20the%20great%20apes%2C%20the,hierarchies%20and%20problem%2Dsolving%20skills.

*NCDs – non-communicable diseases
